# ARID1A-dependent maintenance of H3.3 is required for repressive CHD4-ZMYND8 chromatin interactions at super-enhancers

**DOI:** 10.1186/s12915-022-01407-y

**Published:** 2022-09-25

**Authors:** Jake J. Reske, Mike R. Wilson, Brooke Armistead, Shannon Harkins, Cristina Perez, Joel Hrit, Marie Adams, Scott B. Rothbart, Stacey A. Missmer, Asgerally T. Fazleabas, Ronald L. Chandler

**Affiliations:** 1 Department of Obstetrics, Gynecology and Reproductive Biology, College of Human Medicine, Michigan State University, Grand Rapids, MI 49503 USA; 2 Department of Epigenetics, Van Andel Research Institute, Grand Rapids, MI 49503 USA; 3 Genomics Core Facility, Van Andel Research Institute, Grand Rapids, MI 49503 USA; 4 Department of Women’s Health, Spectrum Health System, Grand Rapids, MI 49341 USA

**Keywords:** ARID1A, SWI/SNF, H3.3, NuRD, Variant histone, Chromatin, Gene expression, Remodeling, Endometrium, Endometrioma

## Abstract

**Background:**

SWI/SNF (BAF) chromatin remodeling complexes regulate lineage-specific enhancer activity by promoting accessibility for diverse DNA-binding factors and chromatin regulators. Additionally, they are known to modulate the function of the epigenome through regulation of histone post-translational modifications and nucleosome composition, although the way SWI/SNF complexes govern the epigenome remains poorly understood. Here, we investigate the function of ARID1A, a subunit of certain mammalian SWI/SNF chromatin remodeling complexes associated with malignancies and benign diseases originating from the uterine endometrium.

**Results:**

Through genome-wide analysis of human endometriotic epithelial cells, we show that more than half of ARID1A binding sites are marked by the variant histone H3.3, including active regulatory elements such as super-enhancers. ARID1A knockdown leads to H3.3 depletion and gain of canonical H3.1/3.2 at ARID1A-bound active regulatory elements, and a concomitant redistribution of H3.3 toward genic elements. ARID1A interactions with the repressive chromatin remodeler CHD4 (NuRD) are associated with H3.3, and ARID1A is required for CHD4 recruitment to H3.3. ZMYND8 interacts with CHD4 to suppress a subset of ARID1A, CHD4, and ZMYND8 co-bound, H3.3+ H4K16ac+ super-enhancers near genes governing extracellular matrix, motility, adhesion, and epithelial-to-mesenchymal transition. Moreover, these gene expression alterations are observed in human endometriomas.

**Conclusions:**

These studies demonstrate that ARID1A-containing BAF complexes are required for maintenance of the histone variant H3.3 at active regulatory elements, such as super-enhancers, and this function is required for the physiologically relevant activities of alternative chromatin remodelers.

**Supplementary Information:**

The online version contains supplementary material available at 10.1186/s12915-022-01407-y.

## Background

The SWI/SNF complex remodels chromatin through ATP-dependent DNA sliding, H2A/H2B dimer eviction, and nucleosome ejection functions [1–3]. SWI/SNF remodeling activities open chromatin and promote accessibility for other DNA-binding factors and chromatin regulators [4, 5]. SWI/SNF complex composition is heterogeneous and cell type dependent [6]. SWI/SNF regulates lineage-specific enhancer activity through multiple mechanisms [7]. Protein subunit architecture contributes to SWI/SNF complex specificity through specialized cofactor interactions. The activities of chromatin remodelers and associated machinery are known to modulate the epigenome by regulating histone post-translational modifications and nucleosome composition [5]. Multiple chromatin remodeler complexes are often observed at the same genomic loci and can perform redundant, cooperative, or antagonistic transcriptional regulatory roles [8].

Subunits within the mammalian SWI/SNF (BAF) chromatin remodeler complex are mutated across an estimated 20% of all human cancers [9]. Tissue-specific propensities for mutations in certain SWI/SNF subunits are also evident [10]. ARID1A (BAF250A) is the most frequently mutated SWI/SNF subunit [11]. ARID1A is the largest SWI/SNF subunit and acts as a structural scaffold for other subunits in certain SWI/SNF complexes [12, 13]. ARID1A also exhibits essential DNA-binding activity albeit in a non-sequence-specific manner [14, 15]. Defects in chromatin accessibility and higher-order chromatin structure are thought to underlie ARID1A and SWI/SNF mutant pathogenesis at least partially [16, 17]. Uterine endometrial cancer displays high rates of ARID1A mutation, with roughly 40% of cases showing loss of ARID1A expression [18, 19]. ARID1A mutations and loss of expression are also observed in deeply invasive forms of endometriosis, which is characterized by ectopic spread of the endometrium [20–22]. ARID1A mutations are also common in endometriosis-associated ovarian cancers [23, 24].

In the endometrial epithelium, we have previously shown that ARID1A normally promotes epithelial identity by repressing the expression of mesenchymal and invasion genes, through promoter-proximal and distal chromatin interactions that affect transcriptional activity [25–27]. In particular, we have found that ARID1A-dependent repression of super-enhancer activity plays critical roles in the maintenance of epithelial identity in the endometrium [25–27]. Other reports have demonstrated that ARID1A and SWI/SNF can function as a repressor, often through interactions with repressive machinery [15, 28–30]. Although nucleosome structure and histone post-translational modifications are suspected mechanisms, it remains poorly understood how SWI/SNF governs the epigenome.

Here, we reveal a mechanism by which ARID1A maintains histone variant H3.3 in active chromatin. This regulation is required for binding of the SWI/SNF-like CHD4 (NuRD) remodeler complex and linked to the CHD4-interacting multivalent histone reader ZMYND8, notably at a subset of super-enhancers. We finally reveal that this mechanism of ARID1A, H3.3, CHD4, and ZMYND8 co-repression targets physiologically relevant genes involved in epithelial-to-mesenchymal transition (EMT) and cellular invasion, and these genes are aberrantly upregulated in human endometriomas. Altogether, our studies reveal a role for ARID1A-containing SWI/SNF complexes in the maintenance of H3.3, and, at a subset of physiologically relevant target genes, H3.3, ARID1A, CHD4, and ZMYND8 are required for transcriptional repression.

## Results

### ARID1A regulates H3.3-associated active chromatin

Our previous studies have demonstrated that ARID1A promotes epithelial characteristics in immortalized 12Z human endometriotic epithelial cells at both the transcriptional and phenotypic levels, such that ARID1A loss leads to epithelial-to-mesenchymal transition (EMT) and enhanced migration and invasion [25, 27]. ARID1A loss in 12Z recapitulates many of the molecular and cellular features observed in ARID1A-deficient endometrial epithelia in vivo [25, 27]. Altogether, 12Z cells represent a model system to explore physiological roles for ARID1A in epigenomic regulation.

**Fig. 1:**
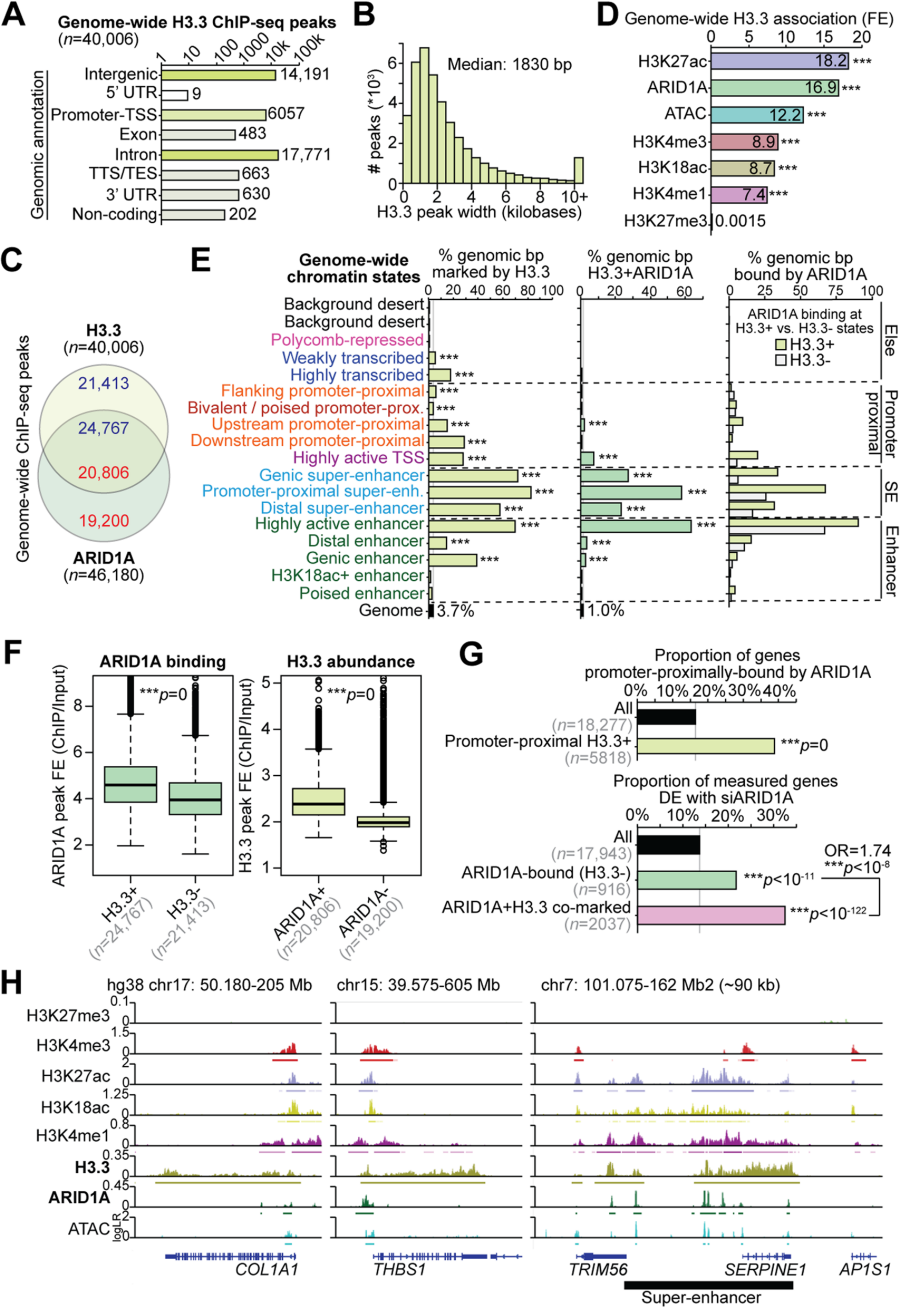
Genome-wide analysis of H3.3-ARID1A chromatin co-regulation. **A** Genomic annotation of 40,006 genome-wide H3.3 ChIP-seq peaks in 12Z cells (*n* = 2). **B** Distribution of H3.3 peak widths. Median H3.3 peak width is 1830 bp. **C** Genome-wide overlap of ARID1A and H3.3 ChIP-seq peaks. **D** Genome-wide association between H3.3 and other previously measured chromatin features, per genomic bp, quantified as [observed / expected]. Statistic is hypergeometric enrichment. **E** Enrichment for H3.3 and ARID1A co-regulation across 18 chromatin states previously modeled via *ChromHMM* [27]. Left, enrichment of H3.3 peaks; center, enrichment of H3.3+ARID1A binding; right, enrichment of ARID1A binding at sites with vs. without H3.3. Statistic is hypergeometric enrichment. **F** Left, ARID1A binding levels (ChIP/input fold-enrichment, FE) at H3.3+ vs. H3.3− ARID1A peaks. Right, H3.3 abundance (ChIP/input fold-enrichment) at ARID1A+ vs. ARID1A− H3.3 peaks. Statistic is two-tailed, unpaired Wilcoxon’s test. **G** Top, enrichment of H3.3 at genes promoter-proximally bound by ARID1A. Bottom, enrichment of ARID1A+H3.3 co-binding at genes DE following ARID1A loss (siARID1A treatment). Statistics are hypergeometric enrichment test and pairwise two-tailed Fisher’s exact test. **H** Example hg38 browser shots of genes and regulatory elements co-regulated by H3.3 and ARID1A. *y*-axis is log-likelihood ratio (logLR) of assay signal (compared to input chromatin for ChIP-seq or background genome for ATAC-seq). Small bars under tracks indicate significant peak detection by *MACS2* (FDR < 0.05). Super-enhancers were detected by *ROSE* from H3K27ac ChIP-seq. * *p* < 0.05, ** *p* < 0.01, *** *p* < 0.001

Histone H3.3 is a variant of canonical H3 with known ties to active chromatin and transcriptional regulation [31, 32]. Like ARID1A, H3.3 has also been observed to mark and regulate active enhancers [33–36]. We investigated the relationship between ARID1A binding and H3.3 in 12Z cells. To measure genome-wide H3.3 localization, we performed H3.3 chromatin immunoprecipitation followed by sequencing (ChIP-seq) in control 12Z cells (*n* = 2 IP replicates). Significant H3.3 enrichment was observed at 40,006 genomic regions (Fig. 1A). Intronic, intergenic, and promoter-TSS regions comprised the vast majority of H3.3 enrichment sites (Fig. 1A). H3.3 ChIP-seq peaks were 1830 bp in width on average and ranged from <500 bp to >10 kilobases (Fig. 1B). Intersecting H3.3 ChIP-seq peaks with our previously published ARID1A ChIP-seq data from these cells [25] revealed that over half of each peak set overlapped (Fig. 1C), a 16.9-fold over-representation genome-wide (Fig. 1D).

We previously constructed a genome-wide chromatin state map accompanying ARID1A loss in 12Z cells via *chromHMM* [37] by measuring seven chromatin features associated with transcriptional regulation: total RNA, ATAC (accessibility), H3K27ac, H3K18ac, H3K4me1, H3K4me3, and H3K27me3 [27]. Similar to our previous reports of ARID1A regulated chromatin states, genomic H3.3 enrichment was highly associated with all active, euchromatic features, but not heterochromatic H3K27me3 (Fig. 1D). Annotating H3.3 enrichment in each of our characterized chromatin states revealed that H3.3 is associated with similar regulatory chromatin states as ARID1A binding, most notably super-enhancers and active typical enhancers (Fig. 1E, left). In agreement, co-regulation by H3.3 and ARID1A was most prominently observed at these same chromatin states (Fig. 1E, center). Next, we examined ARID1A binding at H3.3-marked vs. H3.3-absent chromatin sub-states and found that ARID1A binding was associated with H3.3 at promoter-proximal and genic super-enhancers and active transcription start sites (TSS) (Fig. 1E, right). Upon further investigation of ARID1A and H3.3 genome-wide binding patterns, we observed that genome-wide ARID1A peaks showed overall stronger ARID1A binding when H3.3 was also localized, and H3.3 was overall more abundant at genome-wide H3.3 peaks also bound by ARID1A (Fig. 1F). These data indicate that ARID1A and H3.3 may co-regulate active chromatin elements like enhancers and gene promoters.

We previously reported that ARID1A chromatin binding near gene promoters is associated with transcriptional regulation, such that ARID1A loss leads to aberrant gene expression [25]. Our H3.3 data further revealed that ARID1A binding at promoter-proximal regulatory elements is highly enriched among genes marked by promoter-proximal H3.3 (Fig. 1G, top), indicating that ARID1A transcriptional regulation may be coupled with H3.3. Moreover, the 2037 genes co-marked by ARID1A and H3.3 in the promoter-proximal region (±3 kb surrounding TSS) were more likely to show differential expression (DE) following ARID1A loss than genes without promoter-proximal H3.3 (Fig. 1G, bottom). In addition, locus-scale investigation clearly showed that ARID1A and H3.3 often co-mark active chromatin regulatory elements, which infrequently also includes gene body coating by H3.3, such as at *COL1A1*, *THBS1*, and *SERPINE1* (Fig. 1H). These data collectively suggest H3.3 may be linked to transcriptional regulatory activity by ARID1A at the level of chromatin.

### ARID1A chromatin interactions maintain H3.3

**Fig. 2:**
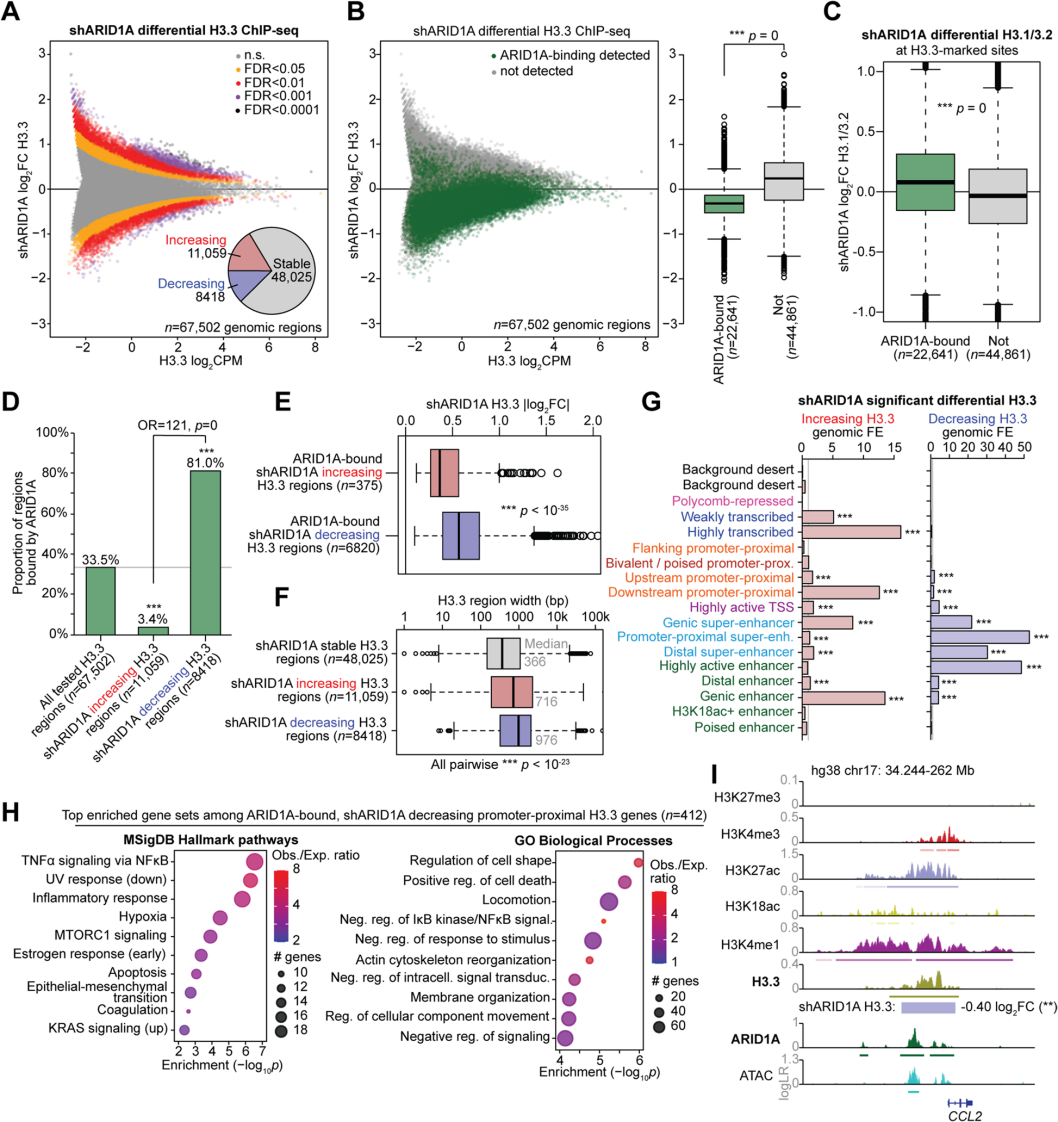
Genome-wide analysis of ARID1A-dependent H3.3. **A** MA plot of shARID1A vs. control differential H3.3 ChIP-seq (*n* = 2), across 67,502 tested genomic regions. Regions are colored based on shARID1A differential H3.3 significance. Inset pie chart depicts distribution of significantly increasing and decreasing H3.3 regions (*csaw*/*edgeR* FDR < 0.05) compared to stable H3.3 (FDR > 0.05). FDR < 0.05 was used as the significance threshold for all downstream analyses. **B** shARID1A differential H3.3 regions segregated by detection of ARID1A binding in wild-type cells. Left, MA plot with all genome-wide H3.3 tested regions, colored by ARID1A binding status. Right, box plot quantification of shARID1A log_2_FC H3.3 abundance, segregated by ARID1A binding status. Statistic is two-tailed, unpaired Wilcoxon’s test. **C** Analysis of canonical H3 (H3.1/3.2) changes (ChIP-seq, *n* = 2) at H3.3-marked genomic regions following ARID1A knockdown (shARID1A), segregated by ARID1A binding status as in **B**. Statistic is two-tailed, unpaired Wilcoxon’s test. **D** Enrichment of ARID1A binding detection at regions with decreasing H3.3 following ARID1A loss compared to all tested H3.3 regions. Statistics are hypergeometric enrichment test and pairwise two-tailed Fisher’s exact test. **E** Magnitude of H3.3 change (log_2_FC) among ARID1A-bound, shARID1A significantly decreasing vs. increasing H3.3 regions. Statistic is two-tailed, unpaired Wilcoxon’s test. **F** Distribution of H3.3-enriched region widths among shARID1A stable vs. increasing vs. decreasing H3.3 regions. Statistic is two-tailed, unpaired Wilcoxon’s test. **G** Chromatin state enrichment among shARID1A increasing and decreasing H3.3 regions, calculated per 200 bp genomic interval. Statistic is hypergeometric enrichment. **H** Top 10 significant (FDR < 0.05) enriched Hallmark pathways (left) and GO Biological Process gene sets (right) among genes with ARID1A-bound, shARID1A decreasing promoter-proximal H3.3. **I** Representative hg38 locus near *CCL2* displaying H3.3 maintained by ARID1A chromatin interactions. *** *p* < 0.001

To understand the relationship between ARID1A and H3.3, we depleted ARID1A from 12Z cells using lentiviral shRNA particles targeting ARID1A (shARID1A) then measured H3.3 by ChIP-seq. Our differential H3.3 ChIP-seq analysis (shARID1A vs. non-targeting shRNA control, *n* = 2) indicated that nearly 1/3 of tested H3.3 regions showed significant differences in H3.3 abundance (*csaw*/*edgeR*, FDR < 0.05) at 72 h following ARID1A knockdown (Fig. 2A, Additional file 1: Fig. S1A). We noted that ARID1A knockdown in 12Z cells did not result in obvious changes in global H3.3 levels by immunoblotting of the histone fraction (Additional file 1: Fig. S1B), suggesting any effects are likely occurring at the level of chromatin. This is further supported by our previously reported 12Z ARID1A knockdown RNA-seq data [25] indicating that the dominantly expressed H3.3-encoding gene isoform, *H3F3B*, does not change in expression (Additional file 1: Fig. S1C). We then investigated how ARID1A chromatin binding may be directly associated with the observed changes in H3.3 following ARID1A loss. Strikingly, ARID1A-bound differential H3.3 regions almost exclusively lost H3.3 and rarely gained H3.3 (Fig. 2B). Corroborating this result, we also profiled canonical H3.1/3.2 histone levels by ChIP-seq (*n* = 2) and observed that ARID1A-bound, H3.3-marked chromatin regions gain H3.1/3.2 (Fig. 2C). While 33% of all tested H3.3 regions had detectable ARID1A binding, 81% of the 8418 shARID1A decreasing H3.3 regions were normally bound by ARID1A, as opposed to only 3% of the 11,059 shARID1A increasing H3.3 regions (Fig. 2D). These results indicate that ARID1A interactions with H3.3 chromatin may serve to promote H3.3 incorporation or maintain its stability. When ARID1A is mutated, H3.3-marked regions shift toward canonical H3.1/3.2.

We further characterized the changes in H3.3 occurring following ARID1A loss. Globally, we found that typical enhancers (distal regions marked by H3K27ac and ATAC, >3 kb away from a TSS and excluding super-enhancers) were enriched for shARID1A-driven H3.3 alterations as compared to gene promoter-proximal regions and super-enhancers (Additional file 1: Fig. S1D). Intriguingly, gene promoter-proximal regions displayed both decreasing and increasing H3.3, whereas distal typical enhancers and super-enhancers almost exclusively lost H3.3 if significantly affected (Additional file 1: Fig. S1E). Among ARID1A-bound genomic H3.3 regions, shARID1A decreasing H3.3 regions tended to display greater differences in H3.3 abundance than shARID1A increasing H3.3 regions (Fig. 2E), supporting a role for ARID1A in promoting maintenance of H3.3 rather than limiting it. Regions that displayed shARID1A decreasing H3.3 also tended to have overall wider genomic footprints than increasing or stable H3.3 regions (Fig. 2F). In agreement with where ARID1A-H3.3 co-regulation is most frequently observed, chromatin state enrichment analysis indicated that ARID1A loss led to depletion of H3.3 at promoter-proximal super-enhancers and highly active enhancers, while increasing H3.3 was observed over actively transcribed gene bodies (Fig. 2G). From the 412 genes we identified with ARID1A-bound, shARID1A decreasing promoter-proximal H3.3, we found significant enrichment for inflammatory, hypoxia, apoptosis, locomotion, and EMT pathways, such as *CCL2* (Fig. 2H,I). These data suggest that ARID1A maintains H3.3 at active regulatory elements such as enhancers and super-enhancers, and, when ARID1A is lost, redistribution of H3.3 occurs toward active genes already marked by H3.3.

### H3.3 depletion phenocopies transcriptional effects of ARID1A loss

**Fig. 3:**
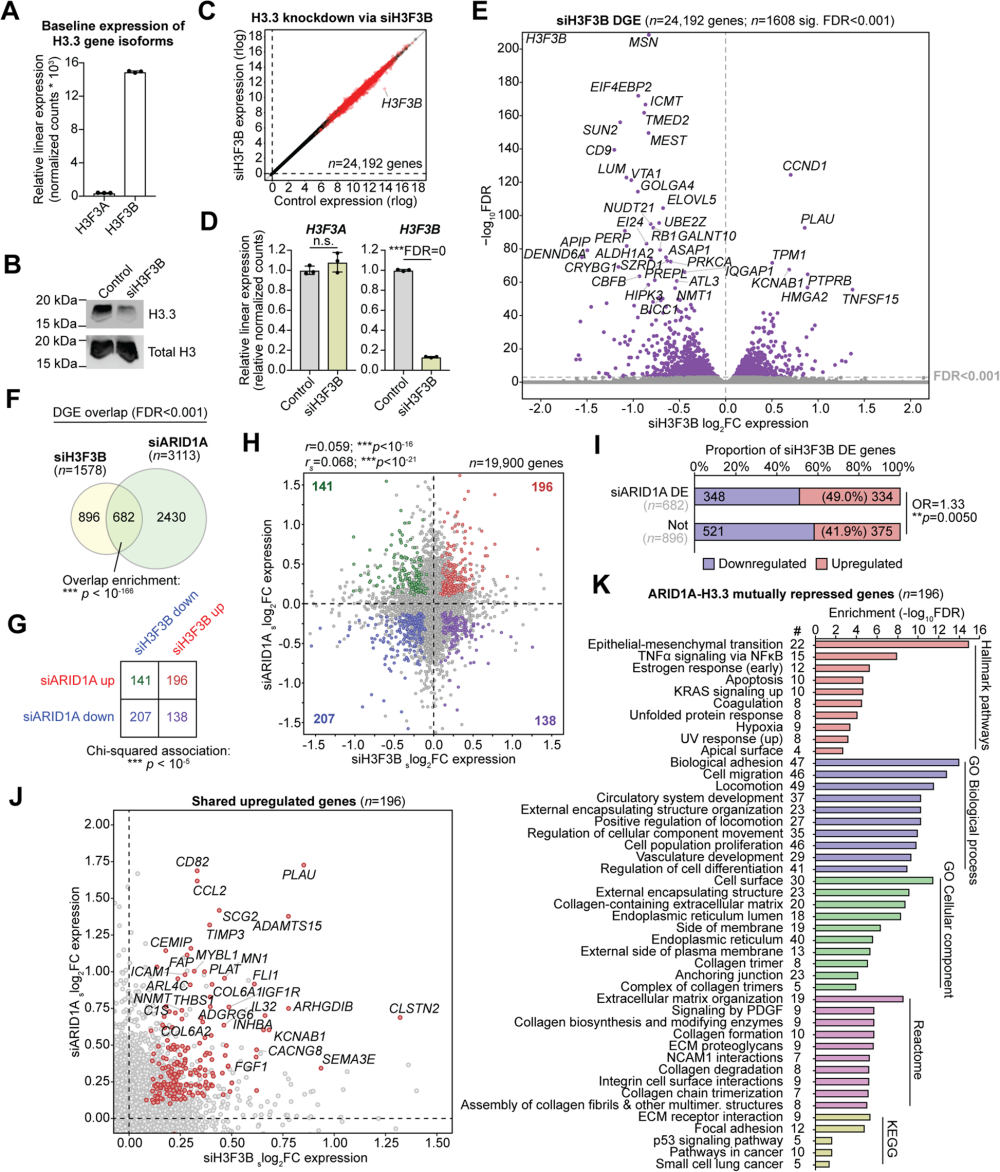
Transcriptional effects of H3.3 depletion and overlap with ARID1A. **A** Baseline relative linear expression of *H3F3A* (*H3-3A*) and *H3F3B* (*H3-3B*) gene isoforms encoding H3.3, as measured by RNA-seq (*n* = 3). **B** Western blot for H3.3 and total H3 in control vs. siH3F3B treated cells. **C** Global transcriptomic effects of 24,192 genes following H3.3 knockdown via siH3F3B treatment (RNA-seq, *n* = 3). Red dots represent significant DE genes (*DESeq2*, FDR < 0.001). **D** Relative linear expression of *H3F3A* and *H3F3B* by RNA-seq in control and siH3F3B cells (*n* = 3). **E** Volcano plot depicting siH3F3B vs. control differential gene expression (DGE). Top significant genes are labeled. **F** Significant overlap in DE genes following H3.3 knockdown (siH3F3B) vs. ARID1A knockdown (siARID1A). Statistic is hypergeometric enrichment. **G** Directional segregation of siH3F3B/siARID1A overlapping DE genes. A positive association is observed by chi-squared test, i.e., genes are more likely to be upregulated or downregulated in both conditions as opposed to antagonistic regulation. **H** Scatter plot of siH3F3B vs. siARID1A expression log_2_FC (with shrinkage correction) for all 19,900 transcriptome-wide commonly detected genes. Statistics are Pearson (*r*) and Spearman (*r*_s_) correlation coefficients. Colored dots indicate significant DE genes (FDR < 0.001) in both treatment conditions. **I** Association between H3.3 transcriptional repression (siH3F3B upregulation) and transcriptional co-regulation by ARID1A (siARID1A DE). Statistic is two-tailed Fisher’s exact test. **J** Scatter plot of 196 shared DE genes upregulated following knockdown of either H3.3 or ARID1A. These genes are mutually repressed by H3.3 and ARID1A. **K** Top significant (FDR < 0.05) enriched gene sets among the 196 ARID1A-H3.3 mutually repressed genes among various gene set databases

We next sought to determine the transcriptional consequences of H3.3 loss in endometrial epithelia. We hypothesized that *H3F3B* could be knocked down to reduce H3.3 levels for acute transcriptome evaluation without impeding cell health (Fig. 3A). Using siRNA targeting *H3F3B* (siH3F3B), we observed H3.3 depletion by immunoblotting without affecting the cell cycle (Fig. 3B, Additional file 1: Fig. S2A-B). RNA-seq transcriptome analysis (*n* = 3) 72 h following siRNA transfection showed clear loss of *H3F3B* expression, but not *H3F3A*, accompanying 1608 significant DE genes (*DESeq2*, FDR < 0.001) including those both upregulated (repressed by H3.3) and downregulated (activated by H3.3) (Fig. 3C–E). As expected, we also observed highly significant enrichment for H3.3-dependent transcriptional changes among genes marked by promoter-proximal H3.3 (Additional file 1: Fig. S2C). Similar to our previous observations with acute ARID1A loss [25], depletion of H3.3 led to mostly minor alterations in gene expression, with the majority of DE genes displaying <0.5 log_2_FC expression change (Fig. 3E). These data indicate H3.3 serves both activating and repressing roles in transcriptional regulation of endometrial epithelial cells.

Comparing the gene expression changes following H3.3 loss with those following ARID1A loss, we observed significant overlap, with 682 shared dysregulated genes (Fig. 3F). These 682 genes were then grouped by direction of change (upregulated vs. downregulated) to identify genes with the same or different expression patterns following ARID1A vs. H3.3 loss. A significant association was observed between the effects of H3.3 and ARID1A loss indicating shared transcriptional consequences (Fig. 3G). Gene expression changes also positively correlated transcriptome-wide (Fig. 3H). Intriguingly, the 682 genes also affected by ARID1A loss were more likely to be transcriptionally repressed by H3.3 (Fig. 3I). In total, 196 genes were identified as mutually repressed by both ARID1A and H3.3, including *PLAU*, *ADAMTS15*, *C1S*, *CD82*, *CCL2*, and *CLSTN2* (Fig. 3J). In agreement with differential H3.3 patterns, these 196 co-repressed genes were enriched for similar gene sets as observed among the ARID1A-bound, shARID1A decreasing promoter-proximal H3.3 gene set, including EMT, TNFα signaling, estrogen response, apoptosis, adhesion, migration, extracellular matrix, and collagens (Fig. 3K). Altogether, these data suggest that ARID1A and H3.3 co-regulate similar target genes in endometrial epithelial cells. At the chromatin level, depletion or destabilization of H3.3 as a result of ARID1A loss may lead to the upregulation of a physiologically relevant set of EMT and invasion genes.

### ARID1A co-regulates H3.3 with CHD4 and ZMYND8

**Fig. 4:**
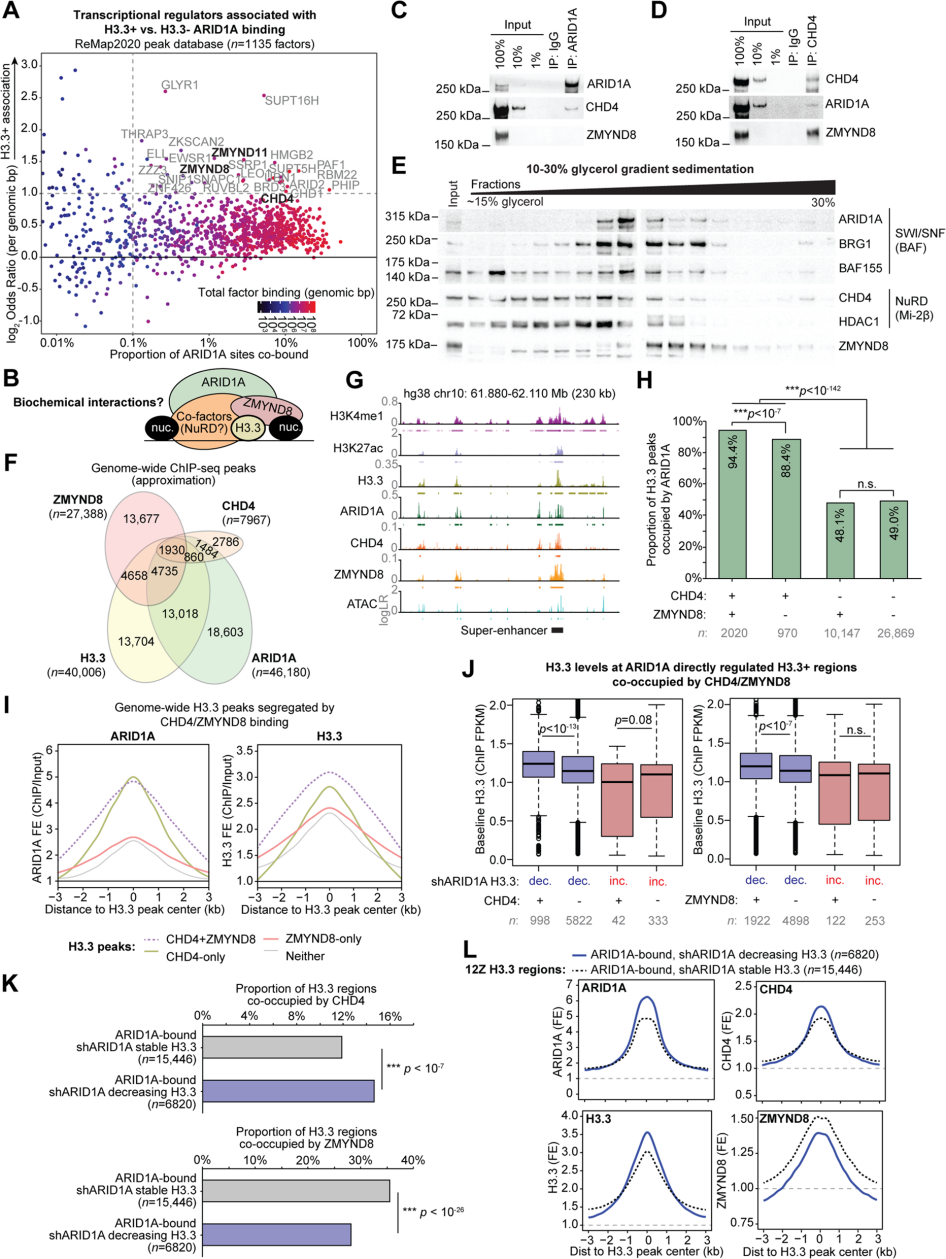
Characterization of ARID1A, CHD4, and ZMYND8 chromatin interactions co-regulating H3.3. **A** Genome-wide associations between ARID1A binding at H3.3+ vs. H3.3− regions for all 1135 transcriptional regulator peak sets included in the ReMap2020 peak database. Labeled factors exhibit an H3.3+ ARID1A binding association with genomic odds ratio >2 and overlap with >0.1% of ARID1A binding sites. ZMYND11 and ZMYND8 (bolded) are two of the top factors most associated with H3.3+ ARID1A binding. **B** Chromatin model schematic depicting hypothesized relationship between ARID1A-SWI/SNF and ZMYND8 co-regulation of H3.3, possibly mediated by co-factors. **C** ARID1A co-immunoprecipitation detecting physical interaction with NuRD catalytic subunit CHD4, but not ZMYND8. **D** CHD4 co-immunoprecipitation detecting physical interactions with both ARID1A and ZMYND8. **E** 10–30% glycerol gradient sedimentation and immunoblotting for SWI/SNF, NuRD, and ZMYND8. Relative fractions display native protein complexes transitioning from low molecular weight (left) to high molecular weight (right). Underlined fractions highlight potential interacting native complexes containing ZMYND8 and members of SWI/SNF (BAF) and NuRD (Mi-2β). **F** Genome-wide ChIP-seq (*n* = 2) peak overlaps between ARID1A, CHD4, ZMYND8, and H3.3. Peak numbers within the Euler diagram are approximations and not mutually exclusive due to varying peak sizes. **G** Example locus on chromosome 10 displaying ARID1A, CHD4, ZMYND8, and H3.3 co-regulation. **H** Enrichment for ARID1A co-regulation of H3.3 peaks bound by CHD4 and/or ZMYND8. Statistic is two-tailed Fisher’s exact test. **I** Average ChIP-seq signal density histograms for ARID1A (left) and H3.3 (right) at H3.3 peaks bound by CHD4 and/or ZMYND8. **J** H3.3 abundance (ChIP FPKM) at ARID1A-bound shARID1A differential H3.3 regions co-bound by CHD4 or ZMYND8. Statistic is two-tailed, unpaired Wilcoxon’s test. **K** Positive association between CHD4 binding (top) and negative association between ZMYND8 binding (bottom) and ARID1A maintenance of H3.3 chromatin, genome-wide. Statistic is two-tailed Fisher’s exact test. **L** Average ChIP-seq signal density histograms for ARID1A, H3.3, CHD4, and ZMYND8 across ARID1A-bound H3.3 regions that decreased or were stable with shARID1A

While our data implicate H3.3 in the ARID1A mutant endometrium, few reports have linked SWI/SNF activity to H3.3 containing nucleosomes [38, 39]. To gain insight into factors associated with H3.3 regulation by ARID1A, we used the ReMap2020 database of 165 million peak regions extracted from genome-wide binding assays [40]. For all 1135 transcriptional regulators included in this database, we calculated genome-wide associations for each set of factor peaks with H3.3-marked (H3.3+) vs. H3.3-absent (H3.3−) ARID1A binding. This analysis revealed that two zinc finger MYND-type proteins, ZMYND11 (BS69) and ZMYND8 (PRKCBP1, RACK7), were among the top co-regulators associated with H3.3+ ARID1A chromatin binding (Fig. 4A, OR = 2.93 and 2.43 for ZMYND11 and ZMYND8, respectively). These data suggest that H3.3 regulation by ARID1A may be mediated by these co-regulators. ZMYND11 and ZMYND8 are multivalent chromatin readers that are suggested to function as interfaces between histones and other chromatin regulator complexes like remodelers, writers, and erasers [41, 42]. Both proteins interact with H3/H4 acetylated tails through bromodomains and may show specificity toward or against H3.3-containing nucleosomes [42–45]. Numerous studies have shown ZMYND8 interacts with and can recruit the SWI/SNF-like repressive NuRD (Mi2-β) chromatin remodeler complex [41, 44, 46, 47], which contains HDACs and interacts with H3.3 [48]. CHD4, a central catalytic subunit of certain NuRD complex configurations, was also associated with H3.3+ ARID1A binding (OR = 1.82) in the ReMap2020 analysis. Like ARID1A, ZMYND8 has also been shown to suppress super-enhancer hyperactivation [49], and, more recently, ZMYND8 and ARID1A were identified in the same screen as key chromatin regulators of EMT [50]. Therefore, we investigated the potential roles of ZMYND8 and possible NuRD co-factors as mediators of the observed ARID1A-H3.3 co-regulation (Fig. 4B).

ARID1A co-immunoprecipitation (co-IP) using an anti-ARID1A antibody was first used to detect physical nuclear interactions with ZMYND8. We previously confirmed the specificity of the anti-ARID1A antibody by co-immunoprecipitation followed by mass spectrometry [25]. While ZMYND8 was not detected in the ARID1A pulldown following high salt washes (300 mM KCl), the NuRD catalytic subunit CHD4 was evident (Fig. 4C). We then hypothesized that CHD4-NuRD may serve as an interface between ARID1A and ZMYND8. A reciprocal CHD4 co-IP confirmed nuclear interactions with both ARID1A and ZMYND8 (Fig. 4D). Glycerol gradient sedimentation revealed native, high molecular weight nuclear fractions containing members of SWI/SNF, NuRD, and ZMYND8 (Fig. 4E). These data suggest that interactions between ARID1A and CHD4 could regulate H3.3 chromatin with support from ZMYND8.

We then examined genome-wide chromatin regulation by CHD4 and ZMYND8 in relation to ARID1A and H3.3. Genome-wide binding profiles of CHD4 and ZMYND8 were measured by ChIP-seq (*n* = 2). Roughly 2000 genomic regions were identified with H3.3 and all three chromatin regulators co-localized (Fig. 4F,G). Across all H3.3 peaks genome-wide, ARID1A binding was most strongly enriched at ZMYND8-CHD4 co-bound sites compared to sites occupied by either CHD4 or ZMYND8 alone (Fig. 4H). Notably, when CHD4 was absent, ARID1A binding at H3.3 sites did not correlate with the presence of ZMYND8 (Fig. 4H). These data suggest that CHD4 may be primarily associated with ARID1A regulation of H3.3 chromatin, while ZMYND8 may be a bystander with unique roles. We further investigated ARID1A binding and H3.3 abundance across H3.3 peaks segregated by the presence of CHD4/ZMYND8. ARID1A binding was again strongest at H3.3 peaks co-bound by CHD4 as opposed to those without CHD4 (Fig. 4I). H3.3 abundance was similarly highest at CHD4-bound peaks, although CHD4+ZMYND8 peaks showed the overall highest H3.3 levels (Fig. 4I). With respect to H3.3 regions dependent on ARID1A chromatin interactions, we observed that baseline H3.3 levels were significantly higher at regions that decreased in H3.3 following ARID1A knockdown if they were co-occupied by CHD4 or ZMYND8, but this was not observed at regions that gained H3.3 following ARID1A loss (Fig. 4J). Intriguingly, we observed that genome-wide H3.3 regions that are normally ARID1A-bound and decrease in H3.3 abundance following ARID1A knockdown are associated with CHD4 but not ZMYND8 (Fig. 4K). Moreover, genome-wide regions that lose H3.3 following ARID1A loss due to disrupted ARID1A chromatin interactions tend to have higher baseline levels of ARID1A, CHD4, and H3.3, but lower levels of ZMYND8 in comparison to stable H3.3 regions (Fig. 4L). Altogether, these results suggest CHD4 and ZMYND8 may be associated with H3.3+ chromatin regulation by ARID1A, but the chromatin logic underlying factor recruitment and functional regulation of H3.3 abundance remains to be elucidated.

### H3.3 maintenance by ARID1A is required for CHD4 binding at a subset of enhancers

Genome-wide binding data indicated that CHD4 is associated with ARID1A regulation of H3.3 chromatin. To further elucidate the role of CHD4 in this process, we first tested whether CHD4 knockdown (shCHD4) also led to changes in genome-wide H3.3 abundance by ChIP-seq (*n* = 2) (Fig. S3A). Differential H3.3 analysis indicated that CHD4 knockdown also led to significant (FDR < 0.05) increasing and decreasing H3.3 abundance across 9331 genomic regions (Fig. S3B), roughly half as many sites as observed following ARID1A loss. However, unlike ARID1A, CHD4 knockdown did not lead to uniform H3.3 depletion at CHD4-bound regions (Fig. S3C). Contrary to ARID1A, we observed a significant enrichment for CHD4 binding at regions that gained H3.3 following CHD4 knockdown, but not those that lost H3.3 (Fig. S3D). Unexpectedly, most sites that displayed changes in H3.3 abundance following CHD4 knockdown did not overlap with those regions changing following ARID1A knockdown (Fig. S3E). Regions that displayed shARID1A decreasing H3.3 were moderately enriched for shCHD4 increasing H3.3, and regions with shARID1A increasing H3.3 were highly enriched for shCHD4 decreasing H3.3 (Fig. S3F-I). Therefore, the effects of CHD4 loss are mostly opposite to the effects of ARID1A loss at the minority of sites where both factors influence H3.3 abundance. Collectively, these results suggest that CHD4 may regulate H3.3 abundance at certain genomic regions, but local CHD4 activity does not appear to be responsible for the observed changes in H3.3 following ARID1A loss.

**Fig. 5:**
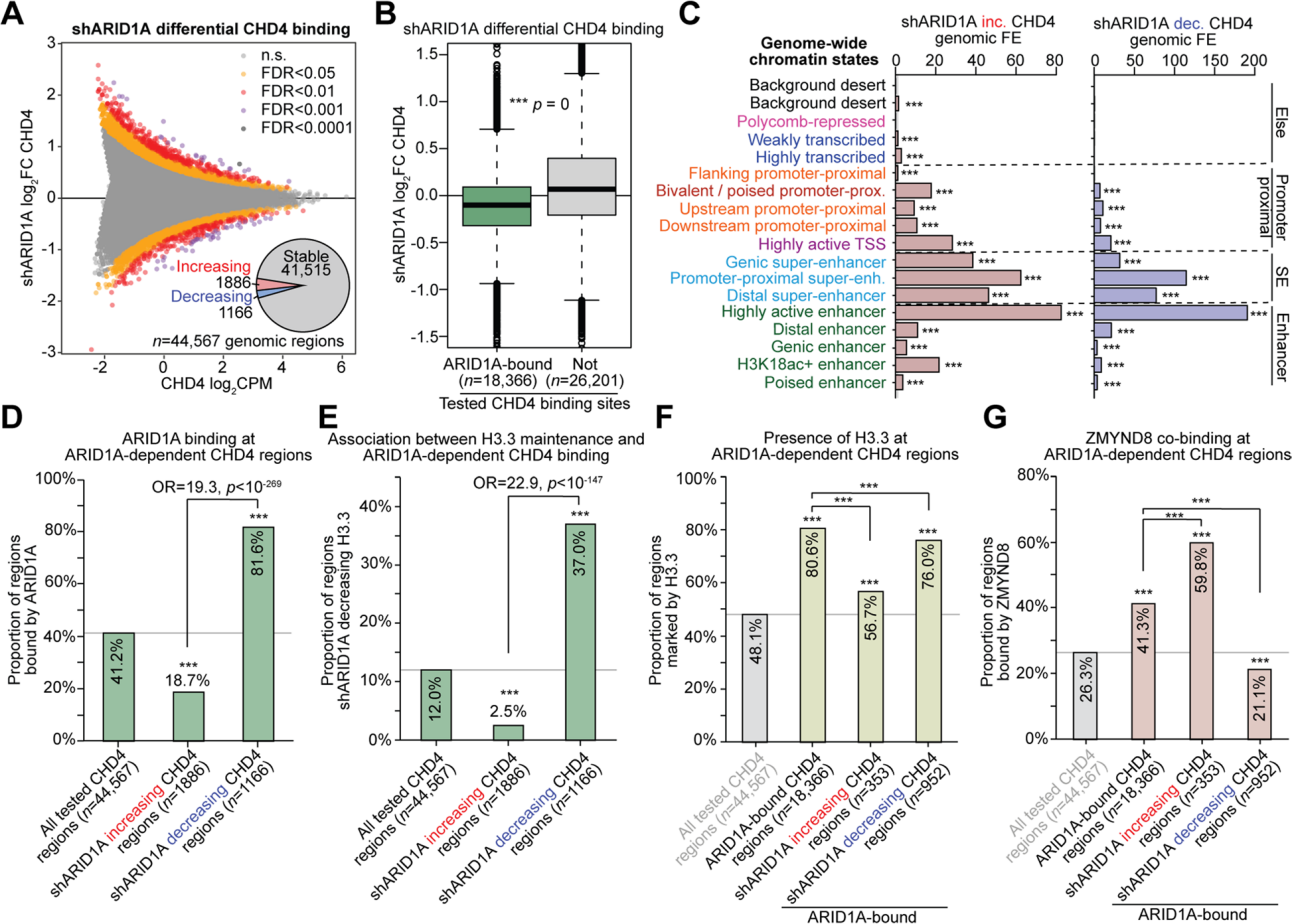
Genome-wide analysis of ARID1A-dependent CHD4 binding. **A** MA plot of shARID1A vs. control differential CHD4 ChIP-seq (*n* = 2), across 44,567 tested genomic regions of CHD4 binding. Regions are colored based on shARID1A differential CHD4 binding significance. Inset pie chart depicts distribution of significantly increasing and decreasing CHD4 regions (*csaw*/*edgeR* FDR < 0.05) compared to stable CHD4 (FDR > 0.05). FDR < 0.05 was used as the significance threshold for all downstream analyses. **B** Global analysis of ARID1A-dependent CHD4 binding based on presence of normal ARID1A binding. Box plot quantification of shARID1A log_2_FC CHD4 binding, segregated by ARID1A binding status. Statistic is two-tailed, unpaired Wilcoxon’s test. **C** Chromatin state enrichment among shARID1A increasing and decreasing CHD4 binding regions, calculated as observed/expected genomic fold-enrichment per genomic bp. Statistic is hypergeometric enrichment. **D** Enrichment of ARID1A binding detection at regions with decreasing CHD4 binding following ARID1A loss compared to all tested CHD4 binding sites. Statistics are hypergeometric enrichment test and pairwise two-tailed Fisher’s exact test. **E** Enrichment of ARID1A-dependent H3.3 maintenance (shARID1A decreasing H3.3 abundance) at regions with decreasing CHD4 binding following ARID1A loss compared to all tested CHD4 binding sites. Statistics are hypergeometric enrichment test and pairwise two-tailed Fisher’s exact test. **F** Associations between presence of H3.3 (wild-type peak) and ARID1A-bound, ARID1A-dependent CHD4 binding. Statistic is hypergeometric enrichment. **G** Associations between ZMYND8 co-binding (wild-type peak) and ARID1A-bound, ARID1A-dependent CHD4 binding. Statistic is hypergeometric enrichment. *** *p* < 0.001

We next examined if CHD4 binding or recruitment may be altered following ARID1A knockdown through ChIP-seq (*n* = 2). Out of 44,567 tested CHD4 binding sites, 1886 regions were detected with significant (FDR < 0.05) increasing CHD4 binding following ARID1A knockdown, and 1166 regions displayed decreased CHD4 binding (Fig. 5A). Strikingly, we also observed a slight global loss of CHD4 binding across ARID1A-bound sites following ARID1A knockdown, as was seen with H3.3 (Fig. 5B). This result suggests that ARID1A may also be required for CHD4 binding at certain genomic regions. Based on our genome-wide chromatin state model, ARID1A knockdown led to CHD4 depletion most specifically at active enhancers and super-enhancers (Fig. 5C). Like H3.3, 81.6% of shARID1A decreasing CHD4 binding regions were normally bound by ARID1A, while only 18.7% of shARID1A increasing CHD4 binding regions were ARID1A-bound (OR = 19.3, Fig. 5D). Importantly, shARID1A decreasing H3.3 was highly associated with regions displaying shARID1A decreasing CHD4 but not increasing CHD4 (Fig. 5E). Further analysis indicated that H3.3 is mostly ubiquitous at regions co-bound by CHD4-ARID1A and thus is not a specific mark of ARID1A-dependent CHD4 binding (Fig. 5F). ZMYND8 binding was less frequent at sites displaying ARID1A-dependent CHD4 binding (Fig. 5G). These data suggest that activities of ARID1A-SWI/SNF promote H3.3 incorporation in active chromatin independently of CHD4 and ZMYND8. Rather, CHD4 recruitment appears to be dependent upon ARID1A maintenance of H3.3 at enhancer elements—putatively through known histone H3.3 reader functions of CHD4.

### ARID1A, CHD4, and ZMYND8 suppress hyperactivation of a subset of H3.3-marked super-enhancers

**Fig. 6:**
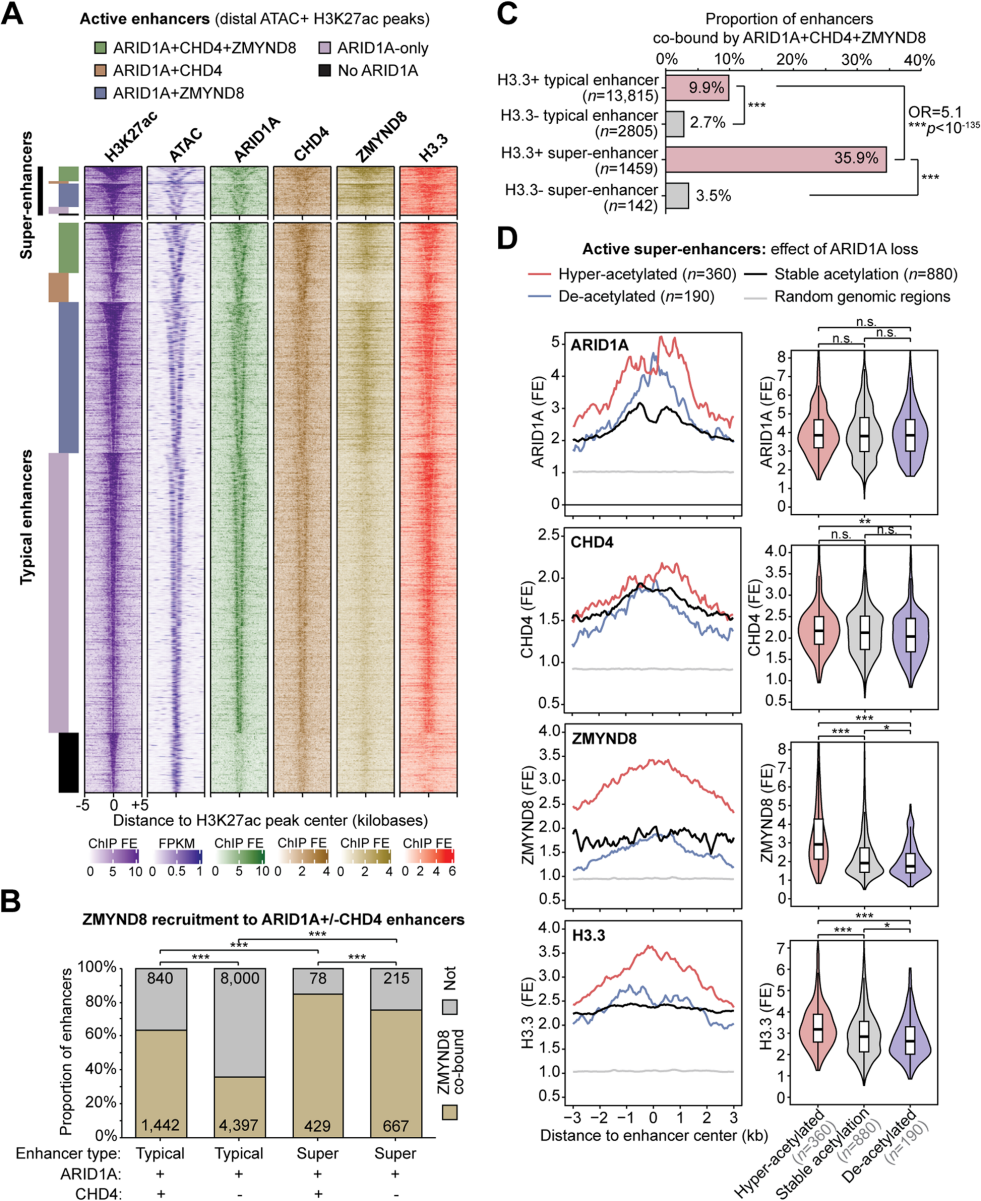
H3.3 enhancer regulation by ARID1A, CHD4, and ZMYND8. **A** Heatmap of chromatin features at 15,925 active typical enhancers and 1374 distal super-enhancer constituents (H3K27ac peaks co-marked by ATAC) segregated by ARID1A ± CHD4 ± ZMYND8 binding. Enhancers are centered on the H3K27ac peak, and signal is displayed as indicated for the flanking 5 kb in either direction. **B** ZMYND8 binding detection at ARID1A-bound typical and super-enhancers with or without CHD4 co-binding. Statistic is two-tailed Fisher’s exact test. **C** Association between ARID1A+CHD4+ZMYND8 co-binding at enhancers and presence of H3.3. H3.3+ super-enhancers show the most frequent co-binding. Statistic is two-tailed Fisher’s exact test. **D** Chromatin features at active super-enhancer constituents segregated by ARID1A loss-driven H3K27-acetylation dynamics: hyperacetylated, de-acetylated, or stably acetylated. Left, average ChIP-seq signal density histograms across enhancer classes. Right, violin plots quantifying signal (ChIP/input fold-enrichment) across enhancer classes. Statistic is two-tailed, unpaired Wilcoxon’s test

Genome-wide analyses indicate that CHD4 often co-binds H3.3+ chromatin in an ARID1A-dependent manner, but the roles of ZMYND8 are unclear and may be chromatin context-specific at subsets of regulatory regions co-bound by all three factors. While ARID1A loss causes widespread H3.3 reduction in chromatin, we observed that ARID1A and H3.3 co-binding is most frequent at active enhancer and super-enhancer chromatin states. As such, we next examined ARID1A, CHD4, and ZMYND8 co-regulation of H3.3 at enhancer elements. At ARID1A-bound active distal enhancers, defined as accessible (ATAC+) H3K27ac peaks located >3 kb from an annotated TSS (Fig. 6A), ZMYND8 binding is associated with presence of CHD4, as expected (Fig. 6B). Moreover, ZMYND8 binding was detected at 84.6% of ARID1A+CHD4 super-enhancers (*n* = 507) as opposed to 63.2% of ARID1A+CHD4 typical enhancers (*n* = 2282) (Fig. 6B). Importantly, ARID1A, CHD4, and ZMYND8 co-binding at active enhancers is associated with presence of H3.3, and this association is greater at super-enhancers than typical enhancers (Fig. 6C). We previously observed that ARID1A suppresses H3K27-hyperacetylation at a subset of active super-enhancers [27]. ARID1A and CHD4 binding levels are not substantially different at suppressed super-enhancers that become hyperacetylated following ARID1A loss vs. those with stable acetylation (Fig. 6D). Strikingly, ZMYND8 binding and H3.3 abundance are significantly higher at suppressed super-enhancers that become hyperacetylated following ARID1A loss, as compared to those with stable or unchanged acetylation (Fig. 6D). These data indicate that ZMYND8 is associated with CHD4 and ARID1A most frequently at active H3.3-marked super-enhancers that are suppressed by ARID1A.

### ZMYND8 is associated with ARID1A and CHD4 chromatin repression toward H4K16ac

**Fig. 7:**
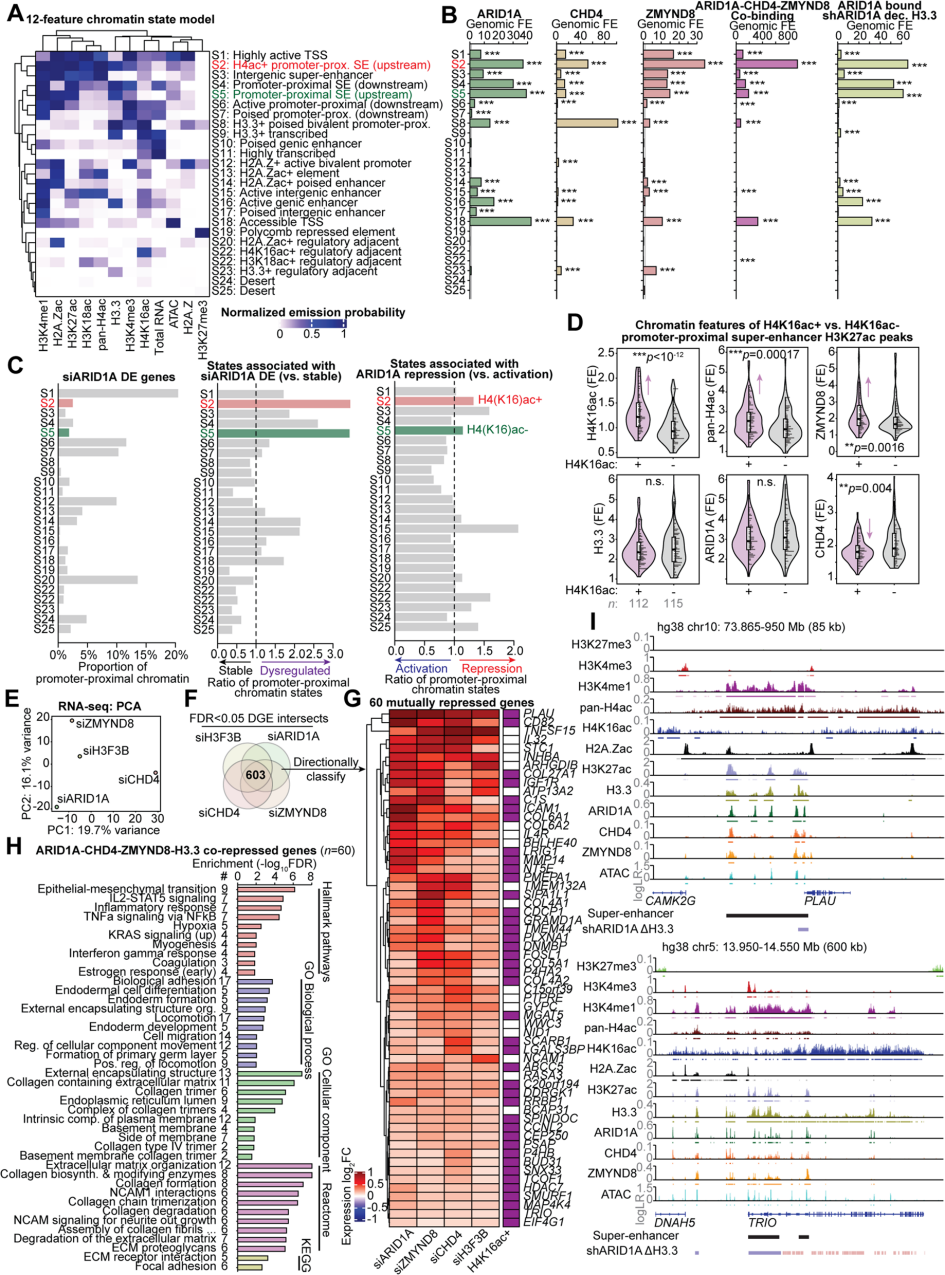
ZMYND8-mediated chromatin repression is associated with H4(K16)ac. **A** Heatmap of clustered, normalized feature emission probabilities, and associated functional annotation of the new 12-feature, genome-wide chromatin 25-state model. States (S_) are labeled based on order of normalized emission probability clustering. See “Methods” for details on optimal model selection. SE: super-enhancer. **B** Genomic fold-enrichment (FE) for ARID1A, CHD4, ZMYND8, co-binding, and shARID1A decreasing H3.3 among the 25 chromatin states. Statistic is hypergeometric enrichment test. **C** Modeled chromatin states among reference gene promoter-proximal regions (±3 kb around annotated TSS). Left, proportion of promoter-proximal chromatin for siARID1A DE genes (*DESeq2*, FDR < 0.0001) belonging to each of the 25 states. Center, ratio of promoter-proximal chromatin states associated with siARID1A DE genes (FDR < 0.0001) compared to stable genes (FDR > 0.05). Right, ratio of promoter-proximal chromatin states associated with ARID1A transcriptional repression (i.e., siARID1A upregulation) compared to activation (i.e., siARID1A downregulation). **D** Violin plots quantifying chromatin feature signal at H4K16ac+ (purple) vs. H4K16ac− (gray) promoter-proximal super-enhancer constituent H3K27ac peaks. Statistic is two-tailed, unpaired Wilcoxon’s test. **E** Principal component analysis (PCA) of RNA-seq expression log_2_FC (shrinkage-corrected) values for siCHD4, siZMYND8, siH3F3B, and siARID1A treatment conditions vs. controls (*n* = 3). In total, 1974 genes with _s_log_2_FC variance >0.1 were used for PCA. **F** Schematic of identifying mechanistic genes co-repressed by ARID1A, H3.3, CHD4, and ZMYND8, i.e., upregulated (*DESeq2*, FDR < 0.05) with siARID1A, siH3F3B, siCHD4, and siZMYND8 treatments. **G** Clustered heatmap of expression log_2_FC values for 60 co-repressed genes upregulated in all 4 knockdown conditions. Rightmost column demarcates presence of H4K16ac peaks over promoter-proximal region or gene body. **H** Top gene sets enriched (hypergeometric enrichment test, FDR < 0.05) among the 60 ARID1A-CHD4-ZMYND8-H3.3 co-repressed genes from various gene set databases. **I** Example target gene loci, *PLAU* and *TRIO*, marked by nearby H3.3+ super-enhancers within H4(K16)ac+ domains that are co-bound by ARID1A, CHD4, and ZMYND8, where ARID1A loss leads to significant depletion of H3.3 (ChIP-seq FDR < 0.05), and knockdown of ARID1A, H3.3, CHD4, or ZMYND8 leads to significant expression upregulation (RNA-seq FDR < 0.05)

Our data indicate the ZMYND8 module appears to be associated H3.3-marked super-enhancers that are repressed by ARID1A. Histone tail reader functions of ZMYND8 are a plausible mechanism through its BRD, PWWP, and PHD domains, which interact with acetylated H3/H4 residues and methylated H3 residues [41]. Particularly, the ZMYND8 bromodomain was recently described to interact with acetylated H4 tails and recruit CHD4 to repress transcription following DNA damage [46]. However, NuRD also interacts with other histone substrates such as H2A.Z, which has been linked to H3.3-mediated transcriptional poising [33]. Of further relevance, P300 was recently shown to acetylate H2A.Z when stimulated by recognition of H4 acetylated residues through its bromodomain [51]. To better resolve our model of how ARID1A, CHD4, and ZMYND8 mediate H3.3 chromatin repression, we generated a more comprehensive genome-wide chromatin state model that contains 5 additional features related to reported ZMYND8 and NuRD activity that were profiled both before and after ARID1A knockdown: H3.3, pan-acetyl-H4 (K5/K8/K12/K16; pan-H4ac), H4K16ac, H2A.Z, and acetyl-H2A.Z (K4/K7; H2A.Zac) (Fig. 7A, Additional file 1: Fig. S4). Since the anti-H2A.Zac antibody (D3V1I, Cell Signaling) used in our assays has not been characterized for specificity toward acetylated H2A.Z containing peptides, we performed a histone peptide array [52] and found that it specifically recognizes H2A.Zac (Additional file 1: Fig. S5). The new 12-feature chromatin state model was quantitatively optimized at 25 chromatin states (see “Methods” for details) (Additional file 1: Fig. S6). The enhanced resolution of our model revealed new state identities such as H3.3+ poised bivalent promoter-proximal elements (state 8), H2A.Zac+ poised enhancers (state 14), and notably the segregation of upstream active promoter-proximal super-enhancers into H4(K16)ac+ (state 2) and negative (state 5) classes (Fig. 7A). We next determined states enriched for co-binding of ARID1A, CHD4, and ZMYND8 and ARID1A-bound, shARID1A decreasing H3.3 (Fig. 7B). Both upstream active promoter-proximal super-enhancer states (states 2 and 5) showed the strongest enrichment for ARID1A-bound, shARID1A decreasing H3.3, while ZMYND8 binding and ARID1A-CHD4-ZMYND8 co-binding was most enriched at the H4(K16)ac+ upstream active promoter-proximal super-enhancer class (state 2) (Fig. 7B).

We further investigated chromatin state identities at reference annotated gene promoter-proximal regions (±3 kb flanking TSS). As expected, the highly active TSS state (state S1) is the most prevalent promoter-proximal chromatin state identity at genes transcriptionally regulated by ARID1A (siARID1A DE genes), while upstream active promoter-proximal super-enhancer states (states 2 and 5) are relatively rare (Fig. 7C, left). However, comparing the promoter-proximal chromatin state identities across siARID1A DE vs. stable genes revealed that upstream active promoter-proximal super-enhancer states are associated with ARID1A transcriptional regulation (Fig. 7C, center). We further segregated promoter-proximal chromatin based on genes that are upregulated with siARID1A (i.e., repressed by ARID1A) vs. downregulated with siARID1A (i.e., activated by ARID1A). The H4(K16)ac+ active promoter-proximal super-enhancer state marked by high ARID1A-CHD4-ZMYND8 co-binding (state 2) showed stronger enrichment for ARID1A transcriptional repression than those without H4(K16)ac (state 5) (Fig. 7C, right). This supports a role for ZMYND8 in specifying transcriptional repression. In agreement, we also observed that chromatin accessibility suppressed by ARID1A chromatin interactions is associated with presence of H4 acetylation (Additional file 1: Fig. S7). Promoter-proximal super-enhancer H3K27ac peaks were next directly segregated by detection of H4K16ac (H4K16ac+, *n* = 112; H4K16ac−, *n* = 115). ARID1A binding and H3.3 abundance were not significantly different between H4K16ac stratified super-enhancers, but ZMYND8 binding was stronger at the H4K16ac+ regions, while CHD4 binding was lower at H4K16ac+ regions (Fig. 7D). Further, a correlation of measured chromatin features across all 227 promoter-proximal super-enhancer constituent enhancers supported that ZMYND8 binding is associated with acetylated H4 marks (Additional file 1: Fig. S8). These analyses collectively suggest that H4(K16)ac-marked promoter-proximal super-enhancers may recruit repressive ZMYND8 to co-regulate a subset of H3.3 marked sites, which are also bound by CHD4 and ARID1A.

To identify genes targeted by repressive regulation of H3.3 chromatin associated with ZMYND8, CHD4, and ARID1A, we also used siRNA to deplete CHD4 (siCHD4) and ZMYND8 (siZMYND8) followed by RNA-seq (*n* = 3) (Fig. 7E, Additional file 1: Fig. S9A-F). As expected, we observed enrichment of expression alterations following loss of ZMYND8 and CHD4 among genes with detected promoter-proximal binding by each factor (Additional file 1: Fig. S9G-H). To further support whether ZMYND8 is associated with transcriptional repression by ARID1A and H3.3, we investigated ZMYND8-mediated gene expression at siARID1A/siH3F3B DGE directional classes (refer to Fig. 3G,H). Indeed, siZMYND8 gene expression alterations were strongly enriched at genes mutually repressed by ARID1A and H3.3 compared to other gene classes, where ZMYND8 also functioned mostly as a repressor (Additional file 1: Fig. S9I).

Differential gene expression again revealed highly overlapping genes transcriptionally regulated by ARID1A, CHD4, ZMYND8, and H3.3 (Additional file 1: Fig. S9J-L), including 603 genes affected by each of the four knockdowns (FDR < 0.05) (Fig. 7F, Additional file 1: Fig. S9L). These included 60 genes mutually repressed by ARID1A, CHD4, ZMYND8, and H3.3 (Fig. 7G). These mechanistic co-repressed genes were enriched for EMT, adhesion, development, locomotion, collagens, and extracellular matrix gene sets (Fig. 7H). Further, 68% of these genes were marked by gene body H4K16ac, an enrichment compared to less than half of all expressed genes (Additional file 1: Fig. S10). Two physiologically relevant target genes revealed through integrative epigenomic analysis are *PLAU* and *TRIO*, both of which are located within broad H4K16ac+ domains and near active H3.3+ super-enhancers co-bound by ARID1A, CHD4, and ZMYND8 (Fig. 7I). ARID1A loss leads to decreased promoter-proximal H3.3 abundance and transcriptional hyperactivation of *PLAU* and *TRIO* (Fig. 7I). We also observed that co-knockdown of ARID1A and CHD4 led to increased induction of *PLAU* compared to either knockdown separately (Additional file 1: Fig. S11).

### ARID1A-H3.3 repressed chromatin targets are aberrantly activated in human endometriomas

**Fig. 8:**
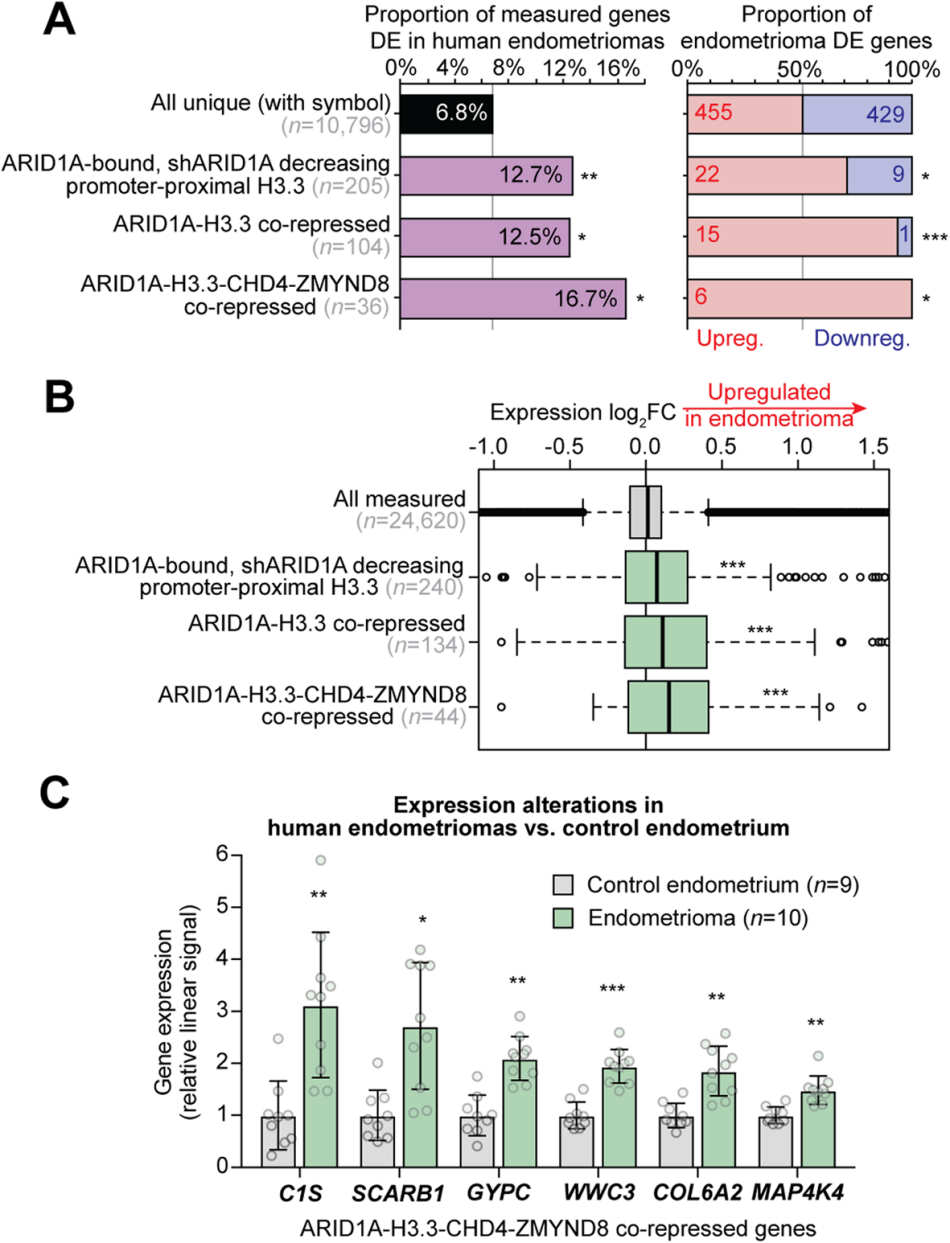
Mechanistic gene expression alterations in human endometriomas. **A** Left, enrichment for ARID1A-H3.3 co-repressive chromatin mechanistic gene sets among human endometrioma (ovarian endometriosis) vs. control endometrium DE genes reported by Hawkins et al. [53], compared to all unique measured genes. Right, proportion of overlapping DE genes that are upregulated vs. downregulated in endometriomas, compared to all unique measured genes. Statistic is hypergeometric enrichment. **B** Box plots displaying endometrioma expression log_2_FC values for probes annotated to genes within mechanistic gene sets, compared to all measured probes. Statistic is two-tailed, unpaired Wilcoxon’s test. **C** Relative expression box-dot plots of 6 genes upregulated in endometriomas vs. control endometrium that are co-repressed by ARID1A, H3.3, CHD4, and ZMYND8. Statistic is *limma* FDR-adjusted *p*. * *p* < 0.05, ** *p* < 0.01, *** *p* < 0.001

**Fig. 9:**
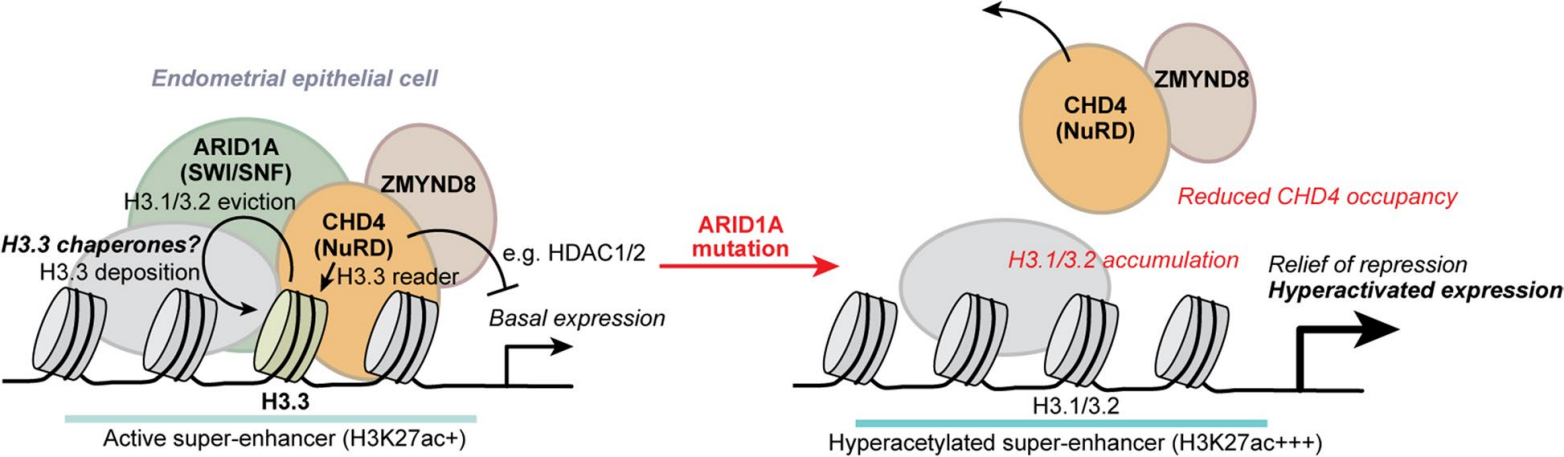
Proposed model of H3.3 chromatin regulation by ARID1A-SWI/SNF and co-regulators. ARID1A and SWI/SNF chromatin remodeling activities are required for H3.3 incorporation or maintenance at certain active regulatory elements across the genome, such as super-enhancers. When ARID1A is mutated or lost, H3.3 maintenance is disrupted, and nucleosome composition shifts toward canonical H3.1/3.2 at ARID1A-bound sites. Consequential to local H3.3 depletion, H3.3 reader factor occupancy is reduced—such as the CHD4-containing NuRD complex—leading to impaired chromatin regulation and aberrant target gene expression. At H3.3+ H4K16ac+ super-enhancer-like elements located promoter-proximally upstream of genes, H3.3 maintenance by ARID1A-SWI/SNF is associated with repression of transcriptional hyperactivation and the NuRD cofactor ZMYND8

Our studies in the 12Z human endometrial epithelial cell line have revealed a mechanism of cooperative regulation by ARID1A, CHD4, and ZMYND8 at H3.3-marked chromatin. To support the relevance of these chromatin regulatory networks on pathologically related gene expression, we utilized a transcriptome expression data set comparing human endometriomas to control endometrial tissue samples [53]. Endometriomas are a result of ectopic spread of endometrial tissue onto the ovary, forming cysts associated with ovarian cancer development [20, 54], and numerous reports have observed high rates of ARID1A mutation or loss of expression in endometriomas [21, 22, 55]. Three ARID1A-H3.3 related gene sets were investigated for relevance in human endometrioma gene expression alterations: (1) ARID1A-bound, shARID1A decreasing promoter-proximal H3.3 genes (*n* = 412), (2) ARID1A-H3.3 co-repressed genes (i.e., siARID1A/siH3F3B upregulated, FDR < 0.001, *n* = 196), and (3) ARID1A-H3.3-CHD4-ZMYND8 co-repressed genes (i.e., upregulated with any knockdown, FDR < 0.05, *n* = 60). We observed significant enrichment for all three of these gene sets among human endometrioma DGE (Fig. 8A, left). Moreover, the overlapping DE genes were more likely to be upregulated in endometriomas than expected by chance, indicating relief of repression is also observed in pathology (Fig. 8A, right). Similarly, examining the endometrioma vs. control endometrium expression log_2_FC values indicated that each gene set tended to be overall upregulated in the pathological, pre-cancerous state (Fig. 8B). Mechanistic genes aberrantly activated in endometriomas that could be attributed to disruption of ARID1A-H3.3 chromatin repression mechanisms include *C1S*, *SCARB1*, *GYPC*, *WWC3*, *COL6A2*, and *MAP4K4* (Fig. 8C). Collectively, our data indicate that ARID1A-SWI/SNF maintains the histone variant H3.3 in active regulatory elements, and a subset of physiologically relevant genes are co-regulated by CHD4 and ZMYND8, such that loss of any of these factors leads to alleviation of transcriptional repression and consequential aberrant gene activation in various endometrial disease contexts where ARID1A mutations are thought to drive pathogenesis (Fig. 9).

## Discussion

We have provided evidence that ARID1A functions to maintain the variant histone H3.3 in active regulatory elements. ARID1A loss leads to H3.3 depletion at active enhancers and super-enhancers, due to disrupted ARID1A chromatin interactions, leading to gain of canonical H3.1/3.2 and redistribution of H3.3 toward active genic and transcribed elements. We further showed that this mechanism is largely independent of H3.3-interacting remodeler CHD4-NuRD. Instead, our data suggest that ARID1A-dependent maintenance of H3.3 is required for CHD4-NuRD binding at a subset of enhancers. Therefore, the BAF complex helps to facilitate H3.3 incorporation, and this activity is required for the recruitment of alternative chromatin remodelers and chromatin regulators with unique regulatory activity.

SWI/SNF is thought to eject nucleosomes and open chromatin [2, 4] rather than assemble nucleosomes. SWI/SNF disruption of nucleosomes may be required for H3.3 incorporation and thus coupled to nucleosome assembly. Therefore, we hypothesize that H3.3 regulation by ARID1A-SWI/SNF occurs by ejecting nucleosomes in favor of H3.3 incorporation by other assembly or chaperone factors, such as HIRA, at active regulatory elements [31]. Unlike the DAXX/ATRX complex, which governs H3.3 incorporation at pericentromeric heterochromatin and telomeres and has intrinsic ATP-dependent remodeling activity, HIRA may rely on other chromatin remodeling complexes for its chaperone activity [31]. In the absence of ARID1A-SWI/SNF, H3.3 nucleosome assembly by HIRA may be impeded by the lack of H3.1/3.2 nucleosome remodeling by the BAF complex. The related CHD1 remodeler is known to be required for H3.3 deposition into chromatin in vivo [56], further suggesting a necessary role for SWI/SNF remodeler activity in H3.3 nucleosome assembly. In addition, both FACT and Polybromo-associated Brm (PBAP) complex are thought to facilitate H3.3 incorporation at boundary elements in *Drosophila* [57]. P400 is another SWI/SNF-like remodeler recently shown to exchange H3.3 nucleosomes that could also possibly collaborate with SWI/SNF [58].

Given previous associations between H3.3 epigenetic memory and cell fate plasticity [32], it is intriguing to consider a role for BAF complex regulation of H3.3 as being a critical determinant of endometrial epithelial cell identity and homeostasis across the menstrual cycle when proliferation and differentiation occur. Further, it remains possible that alternative SWI/SNF complex configurations also participate in H3.3 maintenance, and these complexes could be responsible for H3.3 incorporation at sites unaffected by ARID1A loss.

ARID1A maintenance of H3.3 is associated with genomic interactions with CHD4, a catalytic subunit in the SWI/SNF-like NuRD remodeler complex. As CHD4 knockdown does not lead to the widespread H3.3 depletion observed with ARID1A knockdown, and ARID1A is required for CHD4 recruitment to active regulatory elements, loss of CHD4 co-regulation of H3.3 chromatin is likely the consequence of ARID1A loss. We also observed sub-stoichiometric physical interactions between ARID1A and CHD4, but the significance of direct ARID1A-CHD4 interactions is unclear. CHD4 interactions with histone reader ZMYND8 appear to be associated with further chromatin target regulation specificity, where ZMYND8 may be recruited to H4(K16)ac-marked chromatin through its bromodomain. However, further experimentation, such as ZMYND8 depletion or bromodomain mutation, would be required to confirm the suspected function of the ZMYND8 module in complex recruitment. ZMYND8 co-regulation appears to be associated with chromatin repression, notably at promoter-proximal super-enhancers located upstream of genes, such that disruption of this chromatin mechanism causes relief of repression and subsequent transcriptional hyperactivation. Plasminogen activator urokinase (*PLAU*) was identified as a key target gene repressed by this mechanism in our 12Z endometriotic epithelial cell model. *PLAU* was also recently observed as transcriptionally activated during human menstruation [59], suggesting similar repressive chromatin mechanisms may govern *PLAU* regulation in the healthy endometrium. *PLAU* is upregulated in ovarian endometrioid carcinomas from women with concurrent endometriosis [60], suggesting *PLAU* upregulation may promote malignant transformation in endometriosis. ARID1A mutations are frequently observed in endometriosis-associated ovarian cancers [23, 24]. *C1S*, a component of the complement C1 complex, is another gene that is transcriptionally repressed by ARID1A, CHD4, ZMYND8, and H3.3 that is aberrantly upregulated in human endometriomas. It has been reported that the complement system is activated in women with endometriosis [61], suggesting that ARID1A mutation and associated disruption of chromatin repression may be a possible disease mechanism. In addition to *ARID1A*, it should be noted that *CHD4* mutations leading to nucleosome remodeling defects are also frequent in endometrial cancer [62–64] and may lead to de-repression of similar target genes.

H3.3 is considered an active chromatin mark associated with transcriptional activation. However, our data and others have demonstrated that H3.3 can play roles in transcriptional repression, as well as transcriptional poising and higher-order chromatin regulation, although the mechanisms governing these specific activities remain unclear [31]. A simple hypothesized mechanism explaining how H3.3 can function repressively is through associations with CHD4 and the NuRD complex, as we have studied here. Historically, NuRD has been studied as a repressor due to its subunit composition that includes the histone deacetylases HDAC1/2, although activating roles of NuRD are also known [65–67]. An early study of H3.3 chromatin dynamics indicated that NuRD components were associated with active regions marked by high H3.3 turnover [68]. More recently, NuRD has been shown to directly interact with H3.3 nucleosomes [48]. The finding that CHD4 recruitment is dependent upon H3.3 maintenance by ARID1A at a subset of enhancers further supports the notion that CHD4 co-repressive activity at these sites is likely a result of H3.3 regulation by the BAF complex. When ARID1A is mutated or lost, an H3.3 to H3.1/3.2 switch may impair CHD4 binding through its normal H3.3 reader function, leading to loss of NuRD HDAC co-repressive activity. Intriguingly, CHD4/NuRD was recently shown to control super-enhancer accessibility and maintain lower acetylation levels through its HDAC activity [69], similar to our findings with ARID1A by antagonizing P300 [27]. In support of our data, the authors observed physical interactions between CHD4 and SWI/SNF. Recently, NuRD and SWI/SNF recruitment to active TSS and enhancers was impaired in H3.3K4A mutant mouse ESCs [39], suggesting that NuRD and SWI/SNF recruitment is dependent on the K4 residues on H3.3.

In silico analyses from the ReMap 2020 transcriptional regulator peak database [40] predicted that ZMYND8 is highly associated with H3.3 chromatin regulation by ARID1A. Here, we detected high stringency physical interactions between CHD4 and ZMYND8 as a possible explanation of this co-regulatory activity, as we have demonstrated that ARID1A maintenance of H3.3 is required for CHD4 binding at enhancers. Others have also reported that ZMYND8 interacts with NuRD in numerous contexts [41, 44, 46, 47]. Intriguingly, one recent study reported that ZMYND8 directly recognizes mutant H3.3G34R [70]. Our data indicate that ZMYND8 links repressive H3.3 to H4 acetylation. In support, the ZMYND8 bromodomain directly interacts with acetylated H4 tails [44], and TIP60-mediated H4 acetylation can functionally recruit ZMYND8 through this mechanism to repress transcription with CHD4 in response to DNA damage [46]. Our data also indicate that ARID1A directly suppresses chromatin accessibility at sites marked by H4 acetylation, suggesting that SWI/SNF chromatin remodeler activity may be involved in ZMYND8-NuRD-mediated chromatin repression. ZMYND8-NuRD repression in response to DNA damage was previously shown to rely on KDM5A demethylase activity [71], further suggesting other factors may orchestrate repression vs. activation logic. ZMYND8 has been reported to be a super-enhancer factor that suppresses hyperactivation [49]. Corroborating our results, ZMYND8 was previously shown to associate with NuRD at super-enhancers [47]. We found that super-enhancers that become hyperacetylated following ARID1A loss are normally associated with the highest levels of H3.3 and ZMYND8 binding. In our proposed model, ZMYND8 bromodomain interactions with H4 acetylated tails might facilitate recruitment and transcriptional repression at active chromatin in association with NuRD, such as at H3.3+ super-enhancers. Further work will seek to elucidate how ZMYND8 functions toward transcriptional repression.

In addition to promoter-proximal and distal enhancer chromatin regulation, SWI/SNF, NuRD, and ZMYND8 have been shown to mediate transcriptional pausing and elongation by Pol II and associated machinery [72–75], as well as DNA repair [46, 76, 77]. Super-enhancers mark critical cell identity genes [78], and recent evidence suggests chromatin mechanisms coupling transcription and DNA repair occur at super-enhancers to control transcriptional hyperactivation [79]. Super-enhancer chromatin co-regulation by ARID1A, CHD4, and ZMYND8 may fine-tune transcriptional activation states and thus reflect a mechanism at the intersection of transcriptional regulation and other chromatin-regulated processes.

## Conclusions

In summary, ARID1A-SWI/SNF activities facilitate maintenance of the histone variant H3.3 in active chromatin, such that ARID1A loss leads to local H3.3 depletion, gain of canonical H3.1/3.2, and H3.3 redistribution toward genic elements with transcriptional consequences. At physiologically relevant genomic regions like super-enhancers, ARID1A collaborates with the repressive CHD4-NuRD remodeling complex and reader protein, ZMYND8, to suppress hyperactivation associated with ARID1A-dependent maintenance of H3.3. ARID1A-CHD-ZMYND8-mediated repression affects genes that are aberrantly activated in human endometriomas. These studies have revealed that SWI/SNF regulation of variant histone exchange influences the activities of other chromatin remodelers and regulators by altering nucleosome substrates, and this mechanism plays substantial roles in women’s health and disease.

## Methods

### Cell culture, siRNA transfections, and lentiviral shRNA particle usage

Adherent, human 12Z endometriotic epithelial cells were cultured in DMEM/F12 media in the presence of 10% serum (FBS), 1% L-glutamine, and 1% penicillin/streptomycin. Cells were seeded in antibiotic-free media the day before siRNA transfection. Then, 50 nM siRNA (Dharmacon, ON-TARGETplus) were transfected into cells using the Lipofectamine RNAiMAX (Thermo Fisher Scientific) reagent, according to the manufacturer protocol, in OptiMEM (Gibco). Growth media was replaced 24 h following transfection, without antibiotics. Forty-eight hours after transfection, low serum (0.5% FBS) growth media was added with antibiotics. Cells were harvested 72 h following siRNA transfection. Lentiviral shRNA particles were prepared with Lenti-X 293T cells (Takara) and MISSION pLKO.1 plasmids (Sigma-Aldrich) as previously described [27]. Lentiviral shRNA particles were titered using the qPCR Lentiviral Titration Kit (ABM). shRNA particles were transduced into 12Z cells at a 100-fold multiplicity of infection, and media was replaced 24 h later. Cells were harvested 72 h following shRNA transduction.

### Cell cycle analysis

The Click-iT Plus EdU Flow cytometry Assay Kit (Invitrogen) was used for cell cycle assays. 12Z cells were treated with 10 mM of EdU for 2 h in culture media. Cells were harvested by trypsinization and washed in 1% BSA in PBS. Cells were resuspended in 100 μL of ice-cold PBS, and 900 μL of ice-cold 70% ethanol was added dropwise while vortexing. Cells were incubated on ice for 2 h. Cells were washed with 1% BSA in PBS and then treated with the Click-iT Plus reaction cocktail including Alexa Fluor 488 picolyl azide according to the manufacturer’s instructions for 30 min. Cells were washed with 1× Click-iT permeabilization buffer and wash reagent, and then treated with 5 mM of Vybrant DyeCycle Ruby Stain (Thermo Fisher) diluted in 1% BSA in PBS for 30 min at 37 °C. Flow cytometry was performed using a BD Accuri C6 flow cytometer (BD Biosciences) and analyzed using FlowJo v10 software (BD Biosciences).

### Histone extraction

12Z cells were washed with PBS and scraped in PBS containing 5 mM sodium butyrate. Cells were centrifuged and resuspended in TEB buffer (PBS supplemented with 0.5% Triton X-100, 5 mM sodium butyrate, 2 mM phenylmethylsulfonyl fluoride, 1× protease inhibitor cocktail) and incubated on a 3D spindle nutator at 4 °C for 10 min. Cells were centrifuged at 3000 RPM for 10 min at 4 °C. TEB wash step was repeated once. Following second wash, pellet was resuspended in 0.2 N HCl, and incubated on 3D spindle nutator at 4 °C overnight. The following day, samples were neutralized with 1:10 volume 1M Tris-HCl pH 8.3. Sample was centrifuged at 3000 RPM for 10 min at 4 °C, and supernatant containing histone proteins was collected.

### Co-immunoprecipitation (co-IP)

Nuclear extracts were prepared as previously described [15], dialyzed overnight into 0% glycerol (25 mM HEPES, 0.1 mM EDTA, 12.5 mM MgCl2, 100 mM KCl, 1 mM DTT) using a Slide-A-Lyzer G2 Dialysis Cassette (10 kDa cutoff, Thermo Fisher Scientific), and quantified with the BCA Protein Assay Kit (Pierce, Thermo Fisher Scientific). Primary antibodies (anti-ARID1A, D2A8U, Cell Signaling; anti-CHD4, D8B12, Cell Signaling) were conjugated to Protein A Dynabeads (Invitrogen) overnight at 4 °C in 1× PBS + 0.5% BSA. Normal rabbit IgG (Cell Signaling) IPs were performed in parallel at equivalent masses, as negative controls. Five hundred micrograms nuclear lyase was diluted into IP buffer (20 mM HEPES, 150 mM KCl, 10% glycerol, 0.2 mM EDTA, 0.1% Tween-20, 0.5 mM DTT) to a final volume of 1 mL and clarified by centrifugation. After overnight IP at 4 °C, bead slurries were washed with a series of IP buffers at different KCl concentrations: 2× washes at 150 mM, 3× washes at 300 mM, 2× washes at 100 mM, 1× wash at 60 mM. Immunoprecipitants were eluted in 2× Laemmli buffer + 100 mM DTT at 70 °C for 10 min with agitation.

### Glycerol gradient sedimentation

Nuclear extracts were prepared, dialyzed, and quantified as described in the co-IP methods section. Density sedimentation by glycerol gradient was performed and probed similar to published reports [13]. Briefly, 4.5 mL 10–30% linear glycerol gradients were prepared using an ÄKTA start (Cytiva) from density sedimentation buffer (25 mM HEPES, 0.1 mM EDTA, 12.5 mM MgCl2, 100 mM KCl, 1 mM DTT) additionally containing 30 and 10% glycerol for initial and target concentrations, respectively. Two hundred micrograms nuclear lyase was overlaid on the glycerol gradient followed by ultracentrifugation at 40,000 rpm in an AH-650 swinging bucket rotor (Thermo Fisher Scientific) for 16 h at 4 °C. Two hundred twenty-five microliters gradient fractions were collected and concentrated using StrataClean resin (Agilent). Concentrated fractions were eluted in 1.5× Laemmli buffer + 37.5 mM DTT and run on SDS-PAGE for immunoblotting.

### Immunoblotting

Whole-cell protein lysates were prepared as previously described [27]. Proteins were quantified with the BCA Protein Assay Kit (Pierce, Thermo Fisher Scientific). Protein samples in Laemmli buffer + DTT were denatured at 94 °C for 3 min prior to running on SDS-PAGE gels (6% gels for co-IP and glycerol gradients, 15% gels for histone extracts, and 4–15% gradient gels for whole-cell protein lysates). Gels containing histone extracts were wet transferred to nitrocellulose membranes at 4 °C for 3 h at 400 mA current, then dried at room temperature followed by re-hydration in TBS + 0.1% Tween-20 (TBS-T) and blocking with Odyssey blocking buffer (LI-COR). All other gels were semi-dry transferred to PVDF using a Trans-Blot Turbo (Bio-Rad) according to the manufacturer’s protocol designed for high molecular weight proteins, and blocked with either 5% BSA or 5% milk in TBS. The following primary antibodies were used: anti-ARID1A (D2A8U, Cell Signaling), anti-CHD4 (D4B7, Cell Signaling), anti-ZMYND8 (A302-089, Bethyl), anti-ZMYND8 (Atlas), anti-BRG1 (ab110641, abcam), anti-BAF155 (D7F8S, Cell Signaling), anti-HDAC1 (10E2, Cell Signaling), anti-histone H3.3 (ab176840, abcam), anti-histone H3.3 (2D7-H1, abnova), and anti-histone H3 (D1H2, Cell Signaling). IRDye fluorescent dye (LI-COR) secondary antibodies were used for LI-COR fluorescence-based protein visualization of histones. Horseradish peroxidase (HRP) conjugated secondary antibodies (Cell Signaling) were used for chemiluminescence-based protein visualization of all other targets. Clarity Western ECL substrate (Bio-Rad) was used to activate HRP for chemiluminescence, captured by ChemiDoc XRS+ imaging system (Bio-Rad).

### mRNA-seq and analysis

Seventy-two hours after initial siRNA transfection, and 24 h after low-sera conditioning, 12Z cells were purified for RNA using the Quick-RNA Miniprep Kit (Zymo Research). Transcriptome libraries (*n* = 3 replicates) were prepared and sequenced by the Van Andel Genomics Core from 500 ng of total RNA using the KAPA mRNA HyperPrep kit (v4.17) (Kapa Biosystems). RNA was sheared to 300–400 bp. Prior to PCR amplification, cDNA fragments were ligated to IDT for Illumina unique dual adapters (IDT DNA Inc). Quality and quantity of the finished libraries were assessed using a combination of Agilent DNA High Sensitivity chip (Agilent Technologies), QuantiFluor dsDNA System (Promega), and Kapa Illumina Library Quantification qPCR assays (Kapa Biosystems). Individually indexed libraries were pooled, and 50 bp, paired-end sequencing was performed on an Illumina NovaSeq 6000 sequencer using a 100-cycle sequencing kit (Illumina). Each library was sequenced to an average raw depth of 20–25 million reads. Base calling was done by Illumina RTA3 and output of NCS was demultiplexed and converted to FastQ format with Illumina *Bcl2fastq* v1.9.0.

For analysis, briefly, raw reads were trimmed with *cutadapt* [80] and *Trim Galore!* (http://www.bioinformatics.babraham.ac.uk/projects/trim_galore/) followed by quality control analysis via *FastQC* [81] and *MultiQC* [82]. Trimmed reads were aligned to hg38 assembly and indexed to GENCODE (v28) along with gene feature counting via *STAR* [83]. Low count genes with less than 1 count per sample on average were filtered prior to count normalization and differential gene expression (DGE) analysis by *DESeq2* with empirical Bayes shrinkage for fold-change estimation [84, 85]. Wald probabilities were corrected for multiple testing by independent hypothesis weighting (IHW) [86] for downstream analyses. In presented analyses, “log_2_FC” is the empirically observed log_2_ fold-change in expression between conditions, while “_s_log_2_FC” is a moderated log_2_ fold-change estimate that removes noise from low count genes using the *apeglm* shrinkage estimator as implemented in *DESeq2* [87]. Pairwise comparisons between different DGE analyses and gene sets were initially filtered for genes with transcripts commonly detected in both cell populations.

### Histone peptide arrays

Anti-acetyl-H2A.Z (K4/K7) (D3V1I, Cell Signaling) antibody specificity was analyzed via histone peptide microarrays as previously described [88] with minor modifications. Arrays were designed in *ArrayNinja* [89] and printed using a 2470 Arrayer (Quanterix). All hybridization and wash steps were performed at ambient temperature. Slides were blocked with hybridization buffer (1× PBS [pH 7.6], 0.1% Tween, 5% BSA) for 30 min, then incubated with primary antibody diluted 1:1000 in hybridization buffer for 1 h. Slides were washed 3× for 5 min with PBS, then probed with Alexa647-conjugated secondary antibody diluted 1:5000 in hybridization buffer for 30 min. Slides were washed 3× for 5 min with PBS, dipped in 0.1× PBS to remove salt, and spun dry. Slides were scanned on an InnoScan 1100 microarray scanner (Innopsys), and images were analyzed and quantified using *ArrayNinja*. Plots were generated in Prism (GraphPad). Each peptide antigen is printed six times per array, and each antibody was screened on two separate arrays.

### Chromatin immunoprecipitation (ChIP-seq) and analysis

Wild-type and lentiviral shRNA particle transduced 12Z cells were treated with 1% formaldehyde in growth media for 10 min at ambient temperature. Formaldehyde was quenched by the addition of 0.125 M Glycine and incubation for 5 min at room temperature, followed by PBS wash and scraping. 1×10^7^ crosslinked cells were used for each ChIP, and each antibody and condition for ChIP was performed in duplicate. Chromatin from crosslinked cells was fractionated by digestion with micrococcal nuclease using the SimpleChIP Enzymatic Chromatin IP Kit (Cell Signaling) according to the manufacturer protocol, followed by 30 s of sonication. ChIP was then performed according to the SimpleChIP Enzymatic Chromatin IP Kit (Cell Signaling) with the addition of 5 mM sodium butyrate to preserve histone acetylation. To each 1.25 mL IP, the following antibodies were used: 1:125 anti-histone H3.3 (2D7-H1, abnova); 1:250 anti-histone H3.1/3.2 (61629, Active Motif); 1:50 anti-histone H2A.Z-acetyl (K4/K7) (D3V1I, Cell Signaling); 1:250 anti-histone H2A.Z (ab4174, abcam); 1:50 anti-acetyl-histone H4 (06-866, Millipore); 1:125 anti-histone H4K16ac (39167, Active Motif); 1:50 anti-CHD4 (D4B7, Cell Signaling); 1:250 anti-ZMYND8 (A302-089, Bethyl). Crosslinks were reversed with 0.4 mg/mL Proteinase K (Thermo Fisher) and 0.2 M NaCl at 65 °C for 2 h. DNA was purified using the ChIP DNA Clean & Concentrator Kit (Zymo).

Libraries for input (*n* = 1 per condition) and IP (*n* = 2) samples were prepared by the Van Andel Research Institute Genomics Core. Ten nanograms of material was used for input samples, and the entire precipitated sample was used for IPs. Libraries were generated using the KAPA Hyper Prep Kit (v5.16) (Kapa Biosystems). Prior to PCR amplification, end-repaired and A-tailed DNA fragments were ligated to IDT for Illumina UDI Adapters (IDT DNA Inc.). Quality and quantity of the finished libraries were assessed using a combination of Agilent DNA High Sensitivity chip (Agilent Technologies), QuantiFluor® dsDNA System (Promega), and Kapa Illumina Library Quantification qPCR assays (Kapa Biosystems). Individually indexed libraries were pooled, and 50 bp, paired-end sequencing (for ZMYND8, H3.3, H2A.Zac, and H4K16ac) or 100 bp, single-end sequencing (for CHD4, H2A.Z, and pan-H4ac) was performed on an Illumina NovaSeq 6000 sequencer using a 100-cycle sequencing kit (Illumina). Each library was sequenced to minimum read depth of 80 million reads per input library and 40 million reads per IP library. Base calling was performed by Illumina NCS v2.0, and NCS output was demultiplexed and converted to FastQ format with Illumina *Bcl2fastq* v1.9.0.

New and re-analyzed (differential) ChIP-seq experiments were analyzed as previously described [27]. Briefly, wild-type CHD4 and differential H2A.Z and pan-H4ac ChIP-seq experiments were analyzed as single-end libraries, while wild-type ZMYND8 and differential H3.3, H2A.Zac, and H4K16ac ChIP-seq were analyzed as paired-end libraries. Raw reads for IPs and inputs were trimmed with *cutadapt* [80] and *Trim Galore!* (http://www.bioinformatics.babraham.ac.uk/projects/trim_galore/) followed by quality control analysis via *FastQC* [81] and *MultiQC* [82]. Trimmed reads were aligned to GRCh38.p12 reference genome [90] via *Bowtie2* [91] with flag “--very-sensitive.” Aligned reads were sorted and indexed with *samtools* [92]. Only properly paired read fragments were retained for paired-end libraries via *samtools view* with flag “-f 3” followed by sorting and indexing. For libraries intended for differential analyses, molecular complexity was then estimated from duplicate rates by *ATACseqQC* [93] and *preseqR* [94], and libraries were subsampled to equivalent molecular complexity within an experimental design based on these estimates with *samtools*. *Picard MarkDuplicates* (http://broadinstitute.github.io/picard/) was used to remove PCR duplicates, followed by sorting and indexing. *MACS2* [95] was used to call peaks on each ChIP replicate against the respective input control. For CHD4 and ZMYND8 IPs, *MACS2* called broadPeaks with FDR < 0.05 threshold and otherwise default settings. For H2A.Z and H2A.Zac IPs, *MACS2* called narrowPeaks with FDR < 0.05 threshold and flags “--nomodel --extsize 146” to bypass model building. For H3.3, pan-H4ac, and H4K16ac IPs, MACS2 called broadPeaks with FDR < 0.05 threshold and flags “--nomodel --extsize 146” to bypass model building. The resulting peaks were repeat-masked by ENCODE blacklist filtering and filtered for non-standard contigs [96]. A naive overlapping peak set, as defined by ENCODE [97], was constructed by calling peaks on pooled replicates followed by *bedtools intersect* [98] to select for peaks of at least 50% overlap with each biological replicate.

ChIP-seq differential histone abundance analysis (*n* = 2 per condition) was performed with *csaw* [99]. First, a consensus peak set was constructed for each differential experiment from the union of replicate-intersecting, filtered *MACS2* peak regions called in each condition. When examining the effects of ARID1A knockdown on canonical H3.1/3.2 abundance at H3.3-marked sites, the H3.3 peak set was utilized for this analysis. ChIP reads were counted in these query regions by *csaw*, then filtered for low abundance peaks with average log_2_CPM < −3. When comparing ChIP libraries, any global differences in IP efficiency observed between the two conditions were considered a result of technical bias to ensure a highly conservative analysis. As such, we employed a loess-based local normalization to the peak count matrix, as is implemented in *csaw* [99], to assume a symmetrical MA distribution. A design matrix was then constructed from one “condition” variable. The count matrix and loess offsets were then supplied to *edgeR* [100] for estimating dispersions and fitting quasi-likelihood generalized linear models for differential abundance hypothesis testing. Nearby query regions were then merged up to 500 bp apart for a maximum merged region width of 5 kb, and the most significant probability was used to represent the merged region. Finally, FDR < 0.05 threshold was used to define significant differentially abundant regions.

### Chromatin state modeling and optimization

The same genome-wide chromatin 18-state map of 12Z cells with or without ARID1A depletion, constructed with *ChromHMM* [37, 101] using total RNA, ATAC, H3K4me1, H3K4me3, H3K18ac, H3K27ac, and H3K27me3 data [27], was re-analyzed in Figs. 1, 2, and 5 studies. A refined *ChromHMM* model was constructed with further addition of H3.3, H2A.Z, H2A.Zac (K4/K7), pan-H4ac (K5/K8/K12/K16), and H4K16ac features with some procedural modifications. In order to reduce technical confounders in differential chromatin state analysis between control and ARID1A-depleted cell types, we adopted an equalized binarization framework described by Fiziev et al. [102]. Briefly, the *ChromHMM* chromosomal signal intermediate files during BAM binarization were saved and imported into R. Feature signal values were then background-subtracted by respective control signals when available (e.g., input chromatin for ChIP; does not occur for ATAC). For each feature and cell type, those (background-subtracted) signal values were ranked, and the top *n* ranked binarization calls are selected, where *n* is the lower number of calls among the two cell types for the given feature. The result is a new equalized binarization, where each feature has the same number of “present” region calls in both cell types, per chromosome. As an example, if H3K18ac called 27,000 present regions on chromosome 1 in control cells and 35,000 present regions in ARID1A-depleted cells, then the top 27,000 regions are retained in both cell types. Chromatin state models from 5 to 40 states were then computed using the “concatenated” approach to unify both cell types for differential state comparisons. The new chromatin state model was optimized at 25 states through a strategy devised by Gorkin et al. [103], which utilizes the *ChromHMM CompareModels* function to compare feature emission parameters from the 40-state (most complex) model against all other simpler models, as well as a k-means clustering of emission probabilities from all models together and analyzing the goodness of fit. See Additional file 1: Fig. S6 for related analyses. Across both strategies, 25 states was observed as a threshold for >95% median maximal state correlation and goodness of fit (between-cluster vs. total sum-of-squares) relative to the most complex model.

### Bioinformatics and statistics

The human endometrioma vs. control endometrium genome-wide expression (Illumina BeadChips) data set [53] was retrieved from GEO accession GSE23339 and analyzed via *GEO2R* and *limma* [104–106]. *biomaRt* was used for all gene nomenclature and mouse-human ortholog conversions [107]. The cumulative hypergeometric distribution was calculated in R for enrichment tests. *HOMER* was used to quantify sequencing reads across sets of genomic regions including heatmaps [108]. *GenomicRanges* functions were used to intersect and manipulate genomic coordinates [109]. *IGV* [110] was used for visualizing epigenomic data across hg38 loci as *MACS2* enrichment log-likelihood ratio (logLR) for ChIP-seq and ATAC-seq or FPKM for RNA-seq. Hierarchical clustering by Euclidean distance and heatmaps were generated by *ComplexHeatmap* [111]. *ggplot2* was used for some plots in this study [112]. The statistical language R was used for various computing functions throughout this study [113].

## Supplementary Information

**Additional file 1: Supplementary Information**. **Figure S1**. Supplemental ARID1A knockdown differential H3.3 data. **Figure S2**. H3.3 knockdown functional analysis. **Figure S3**. Effects of CHD4 loss on H3.3 chromatin and comparison to ARID1A. **Figure S4**. Additional chromatin features profiled in 12Z cells. **Figure S5**. Peptide specificity of anti-acetyl-H2A.Z (K4/K7). **Figure S6**. ChromHMM model optimization. **Figure S7**. Chromatin accessibility repressed by ARID1A is associated with H4 acetylation. **Figure S8**. Chromatin feature correlation across promoter-proximal super-enhancers. **Figure S9**. siCHD4/siZMYND8 functional analysis. **Figure S10**. H4K16ac enrichment at repressed mechanistic genes. **Figure S11**. Additive transcriptional repression by ARID1A and CHD4. **Figure S12**. Uncropped Western blots.

## Data Availability

All data generated or analyzed during this study are included in this published article, its supplementary information files and publicly available repositories. The novel data sets supporting the conclusions of this article are available in the Gene Expression Omnibus (GEO, https://www.ncbi.nlm.nih.gov/geo/) at SuperSeries accession GSE190557 [114]. Previously reported data were retrieved from GEO at accessions GSE121198 [115], GSE148474 [116], and GSE23339 [117] and re-analyzed as previously described [25, 27] or as stated in the “Methods” section.

## Abbreviations

ATAC: Assay for Transposase-Accessible Chromatin
ATAC-seq: Assay for Transposase-Accessible Chromatin followed by sequencing
ChIP: Chromatin immunoprecipitation
ChIP-seq: Chromatin immunoprecipitation followed by sequencing
DGE: Differential gene expression
EMT: Epithelial-to-mesenchymal transition
FDR: False discovery rate
FE: Fold-enrichment
GEO: Gene Expression Omnibus
Co-IP: Co-immunoprecipitation
log_2_FC: log_2_ fold-change
logLR: log-likelihood ratio
n.s.: Not significant
*p*: Probability [of null hypothesis]
PCA: Principal component analysis
OR: Odds ratio
SE: Super-enhancer
TSS: Transcription start site

## References

[1] Whitehouse I, Flaus A, Cairns BR, White MF, Workman JL, Owen-Hughes T (1999). Nucleosome mobilization catalysed by the yeast SWI/SNF complex. Nature.

[2] Dechassa ML, Sabri A, Pondugula S, Kassabov SR, Chatterjee N, Kladde MP, Bartholomew B (2010). SWI/SNF has intrinsic nucleosome disassembly activity that is dependent on adjacent nucleosomes. Mol Cell.

[3] Kassabov SR, Zhang B, Persinger J, Bartholomew B (2003). SWI/SNF unwraps, slides, and rewraps the nucleosome. Mol Cell.

[4] Clapier CR, Cairns BR (2009). The biology of chromatin remodeling complexes. Annu Rev Biochem.

[5] Clapier CR. Sophisticated conversations between chromatin and chromatin remodelers, and dissonances in cancer. Int J Mol Sci. 2021;22(11). https://pubmed.ncbi.nlm.nih.gov/34070411/. 10.3390/ijms22115578 PMC8197500 34070411

[6] Wang W, Cote J, Xue Y, Zhou S, Khavari PA, Biggar SR, Muchardt C, Kalpana GV, Goff SP, Yaniv M (1996). Purification and biochemical heterogeneity of the mammalian SWI-SNF complex. EMBO J.

[7] Alver BH, Kim KH, Lu P, Wang X, Manchester HE, Wang W, Haswell JR, Park PJ, Roberts CW (2017). The SWI/SNF chromatin remodelling complex is required for maintenance of lineage specific enhancers. Nat Commun.

[8] Morris SA, Baek S, Sung MH, John S, Wiench M, Johnson TA, Schiltz RL, Hager GL (2014). Overlapping chromatin-remodeling systems collaborate genome wide at dynamic chromatin transitions. Nat Struct Mol Biol.

[9] Kadoch C, Hargreaves DC, Hodges C, Elias L, Ho L, Ranish J, Crabtree GR (2013). Proteomic and bioinformatic analysis of mammalian SWI/SNF complexes identifies extensive roles in human malignancy. Nat Genet.

[10] Kadoch C, Crabtree GR (2015). Mammalian SWI/SNF chromatin remodeling complexes and cancer: mechanistic insights gained from human genomics. Sci Adv.

[11] Mittal P, Roberts CWM (2020). The SWI/SNF complex in cancer - biology, biomarkers and therapy. Nat Rev Clin Oncol.

[12] He S, Wu Z, Tian Y, Yu Z, Yu J, Wang X, Li J, Liu B, Xu Y (2020). Structure of nucleosome-bound human BAF complex. Science.

[13] Mashtalir N, D'Avino AR, Michel BC, Luo J, Pan J, Otto JE, Zullow HJ, McKenzie ZM, Kubiak RL, St Pierre R (2018). Modular organization and assembly of SWI/SNF family chromatin remodeling complexes. Cell.

[14] Dallas PB, Pacchione S, Wilsker D, Bowrin V, Kobayashi R, Moran E (2000). The human SWI-SNF complex protein p270 is an ARID family member with non-sequence-specific DNA binding activity. Mol Cell Biol.

[15] Chandler RL, Brennan J, Schisler JC, Serber D, Patterson C, Magnuson T (2013). ARID1a-DNA interactions are required for promoter occupancy by SWI/SNF. Mol Cell Biol.

[16] Kelso TWR, Porter DK, Amaral ML, Shokhirev MN, Benner C, Hargreaves DC. Chromatin accessibility underlies synthetic lethality of SWI/SNF subunits in ARID1A-mutant cancers. Elife. 2017;6. https://pubmed.ncbi.nlm.nih.gov/28967863/. 10.7554/eLife.30506 PMC5643100 28967863

[17] Barutcu AR, Lajoie BR, Fritz AJ, McCord RP, Nickerson JA, van Wijnen AJ, Lian JB, Stein JL, Dekker J, Stein GS (2016). SMARCA4 regulates gene expression and higher-order chromatin structure in proliferating mammary epithelial cells. Genome Res.

[18] Wu RC, Wang TL, Shih Ie M (2014). The emerging roles of ARID1A in tumor suppression. Cancer Biol Ther.

[19] Kandoth C, Schultz N, Cherniack AD, Akbani R, Liu Y, Shen H, Robertson AG, Pashtan I, Shen R, Cancer Genome Atlas Research N (2013). Integrated genomic characterization of endometrial carcinoma. Nature.

[20] Zondervan KT, Becker CM, Missmer SA (2020). Endometriosis. N Engl J Med.

[21] Samartzis EP, Samartzis N, Noske A, Fedier A, Caduff R, Dedes KJ, Fink D, Imesch P (2012). Loss of ARID1A/BAF250a-expression in endometriosis: a biomarker for risk of carcinogenic transformation?. Mod Pathol.

[22] Anglesio MS, Papadopoulos N, Ayhan A, Nazeran TM, Noe M, Horlings HM, Lum A, Jones S, Senz J, Seckin T (2017). Cancer-associated mutations in endometriosis without cancer. N Engl J Med.

[23] Wiegand KC, Shah SP, Al-Agha OM, Zhao Y, Tse K, Zeng T, Senz J, McConechy MK, Anglesio MS, Kalloger SE (2010). ARID1A mutations in endometriosis-associated ovarian carcinomas. N Engl J Med.

[24] Jones S, Wang TL, Shih Ie M, Mao TL, Nakayama K, Roden R, Glas R, Slamon D, Diaz LA, Vogelstein B (2010). Frequent mutations of chromatin remodeling gene ARID1A in ovarian clear cell carcinoma. Science.

[25] Wilson MR, Reske JJ, Holladay J, Wilber GE, Rhodes M, Koeman J, Adams M, Johnson B, Su RW, Joshi NR (2019). ARID1A and PI3-kinase pathway mutations in the endometrium drive epithelial transdifferentiation and collective invasion. Nat Commun.

[26] Reske JJ, Wilson MR, Holladay J, Wegener M, Adams M, Chandler RL (2020). SWI/SNF inactivation in the endometrial epithelium leads to loss of epithelial integrity. Hum Mol Genet.

[27] Wilson MR, Reske JJ, Holladay J, Neupane S, Ngo J, Cuthrell N, Wegener M, Rhodes M, Adams M, Sheridan R (2020). ARID1A mutations promote P300-dependent endometrial invasion through super-enhancer hyperacetylation. Cell Rep.

[28] Rafati H, Parra M, Hakre S, Moshkin Y, Verdin E, Mahmoudi T (2011). Repressive LTR nucleosome positioning by the BAF complex is required for HIV latency. PLoS Biol.

[29] Van Rechem C, Boulay G, Leprince D (2009). HIC1 interacts with a specific subunit of SWI/SNF complexes, ARID1A/BAF250A. Biochem Biophys Res Commun.

[30] Bui CB, Le HK, Vu DM, Truong KD, Nguyen NM, Ho MAN, Truong DQ (2019). ARID1A-SIN3A drives retinoic acid-induced neuroblastoma differentiation by transcriptional repression of TERT. Mol Carcinog.

[31] Shi L, Wen H, Shi X (2017). The histone variant H3.3 in transcriptional regulation and human disease. J Mol Biol.

[32] Szenker E, Ray-Gallet D, Almouzni G (2011). The double face of the histone variant H3.3. Cell Res.

[33] Chen P, Zhao J, Wang Y, Wang M, Long H, Liang D, Huang L, Wen Z, Li W, Li X (2013). H3.3 actively marks enhancers and primes gene transcription via opening higher-ordered chromatin. Genes Dev.

[34] Deaton AM, Gomez-Rodriguez M, Mieczkowski J, Tolstorukov MY, Kundu S, Sadreyev RI, et al. Enhancer regions show high histone H3.3 turnover that changes during differentiation. Elife. 2016;5. https://pubmed.ncbi.nlm.nih.gov/27304074/. 10.7554/eLife.15316 PMC4965263 27304074

[35] Martire S, Gogate AA, Whitmill A, Tafessu A, Nguyen J, Teng YC, Tastemel M, Banaszynski LA (2019). Phosphorylation of histone H3.3 at serine 31 promotes p300 activity and enhancer acetylation. Nat Genet.

[36] Zhang H, Gan H, Wang Z, Lee JH, Zhou H, Ordog T, Wold MS, Ljungman M, Zhang Z (2017). RPA interacts with HIRA and regulates H3.3 deposition at gene regulatory elements in mammalian cells. Mol Cell.

[37] Ernst J, Kellis M (2012). ChromHMM: automating chromatin-state discovery and characterization. Nat Methods.

[38] Pillidge Z, Bray SJ. SWI/SNF chromatin remodeling controls Notch-responsive enhancer accessibility. EMBO Rep. 2019;20(5). https://pubmed.ncbi.nlm.nih.gov/30914409/. 10.15252/embr.201846944 PMC6500979 30914409

[39] Gehre M, Bunina D, Sidoli S, Lubke MJ, Diaz N, Trovato M, Garcia BA, Zaugg JB, Noh KM (2020). Lysine 4 of histone H3.3 is required for embryonic stem cell differentiation, histone enrichment at regulatory regions and transcription accuracy. Nat Genet.

[40] Cheneby J, Menetrier Z, Mestdagh M, Rosnet T, Douida A, Rhalloussi W, Bergon A, Lopez F, Ballester B (2020). ReMap 2020: a database of regulatory regions from an integrative analysis of human and Arabidopsis DNA-binding sequencing experiments. Nucleic Acids Res.

[41] Savitsky P, Krojer T, Fujisawa T, Lambert JP, Picaud S, Wang CY, Shanle EK, Krajewski K, Friedrichsen H, Kanapin A (2016). Multivalent histone and DNA engagement by a PHD/BRD/PWWP triple reader cassette recruits ZMYND8 to K14ac-rich chromatin. Cell Rep.

[42] Guo R, Zheng L, Park JW, Lv R, Chen H, Jiao F, Xu W, Mu S, Wen H, Qiu J (2014). BS69/ZMYND11 reads and connects histone H3.3 lysine 36 trimethylation-decorated chromatin to regulated pre-mRNA processing. Mol Cell.

[43] Wen H, Li Y, Xi Y, Jiang S, Stratton S, Peng D, Tanaka K, Ren Y, Xia Z, Wu J (2014). ZMYND11 links histone H3.3K36me3 to transcription elongation and tumour suppression. Nature.

[44] Adhikary S, Sanyal S, Basu M, Sengupta I, Sen S, Srivastava DK, Roy S, Das C (2016). Selective recognition of H3.1K36 dimethylation/H4K16 acetylation facilitates the regulation of all-trans-retinoic acid (ATRA)-responsive genes by putative chromatin reader ZMYND8. J Biol Chem.

[45] Li N, Li Y, Lv J, Zheng X, Wen H, Shen H, Zhu G, Chen TY, Dhar SS, Kan PY (2016). ZMYND8 reads the dual histone mark H3K4me1-H3K14ac to antagonize the expression of metastasis-linked genes. Mol Cell.

[46] Gong F, Chiu LY, Cox B, Aymard F, Clouaire T, Leung JW, Cammarata M, Perez M, Agarwal P, Brodbelt JS (2015). Screen identifies bromodomain protein ZMYND8 in chromatin recognition of transcription-associated DNA damage that promotes homologous recombination. Genes Dev.

[47] Spruijt CG, Luijsterburg MS, Menafra R, Lindeboom RG, Jansen PW, Edupuganti RR, Baltissen MP, Wiegant WW, Voelker-Albert MC, Matarese F (2016). ZMYND8 co-localizes with NuRD on target genes and regulates poly(ADP-Ribose)-dependent recruitment of GATAD2A/NuRD to sites of DNA damage. Cell Rep.

[48] Kraushaar DC, Chen Z, Tang Q, Cui K, Zhang J, Zhao K (2018). The gene repressor complex NuRD interacts with the histone variant H3.3 at promoters of active genes. Genome Res.

[49] Shen H, Xu W, Guo R, Rong B, Gu L, Wang Z, He C, Zheng L, Hu X, Hu Z (2016). Suppression of enhancer overactivation by a RACK7-histone demethylase complex. Cell.

[50] Serresi M, Kertalli S, Li L, Schmitt MJ, Dramaretska Y, Wierikx J, et al. Functional antagonism of chromatin modulators regulates epithelial-mesenchymal transition. Sci Adv. 2021;7(9). https://pubmed.ncbi.nlm.nih.gov/33627422/. 10.1126/sciadv.abd7974 PMC7904264 33627422

[51] Colino-Sanguino Y, Cornett EM, Moulder D, Smith GC, Hrit J, Cordeiro-Spinetti E, Vaughan RM, Krajewski K, Rothbart SB, Clark SJ (2019). A read/write mechanism connects p300 bromodomain function to H2A.Z acetylation. iScience.

[52] Rothbart SB, Krajewski K, Strahl BD, Fuchs SM (2012). Peptide microarrays to interrogate the "histone code". Methods Enzymol.

[53] Hawkins SM, Creighton CJ, Han DY, Zariff A, Anderson ML, Gunaratne PH, Matzuk MM (2011). Functional microRNA involved in endometriosis. Mol Endocrinol.

[54] Grandi G, Toss A, Cortesi L, Botticelli L, Volpe A, Cagnacci A (2015). The association between endometriomas and ovarian cancer: preventive effect of inhibiting ovulation and menstruation during reproductive life. Biomed Res Int.

[55] Borrelli GM, Abrao MS, Taube ET, Darb-Esfahani S, Kohler C, Chiantera V, Mechsner S (2016). (Partial) Loss of BAF250a (ARID1A) in rectovaginal deep-infiltrating endometriosis, endometriomas and involved pelvic sentinel lymph nodes. Mol Hum Reprod.

[56] Konev AY, Tribus M, Park SY, Podhraski V, Lim CY, Emelyanov AV, Vershilova E, Pirrotta V, Kadonaga JT, Lusser A (2007). CHD1 motor protein is required for deposition of histone variant H3.3 into chromatin in vivo. Science.

[57] Nakayama T, Shimojima T, Hirose S (2012). The PBAP remodeling complex is required for histone H3.3 replacement at chromatin boundaries and for boundary functions. Development.

[58] Pradhan SK, Su T, Yen L, Jacquet K, Huang C, Cote J, Kurdistani SK, Carey MF (2016). EP400 deposits H3.3 into promoters and enhancers during gene activation. Mol Cell.

[59] Wang W, Vilella F, Alama P, Moreno I, Mignardi M, Isakova A, Pan W, Simon C, Quake SR (2020). Single-cell transcriptomic atlas of the human endometrium during the menstrual cycle. Nat Med.

[60] Zhang C, Wang X, Anaya Y, Parodi L, Cheng L, Anderson ML, Hawkins SM (2018). Distinct molecular pathways in ovarian endometrioid adenocarcinoma with concurrent endometriosis. Int J Cancer.

[61] Sikora J, Wroblewska-Czech A, Smycz-Kubanska M, Mielczarek-Palacz A, Cygal A, Witek A, Kondera-Anasz Z (2018). The role of complement components C1q, MBL and C1 inhibitor in pathogenesis of endometriosis. Arch Gynecol Obstet.

[62] Le Gallo M, O'Hara AJ, Rudd ML, Urick ME, Hansen NF, O'Neil NJ, Price JC, Zhang S, England BM, Godwin AK (2012). Exome sequencing of serous endometrial tumors identifies recurrent somatic mutations in chromatin-remodeling and ubiquitin ligase complex genes. Nat Genet.

[63] Berger AC, Korkut A, Kanchi RS, Hegde AM, Lenoir W, Liu W, Liu Y, Fan H, Shen H, Ravikumar V (2018). A comprehensive pan-cancer molecular study of gynecologic and breast cancers. Cancer Cell.

[64] Kovac K, Sauer A, Macinkovic I, Awe S, Finkernagel F, Hoffmeister H, Fuchs A, Muller R, Rathke C, Langst G (2018). Tumour-associated missense mutations in the dMi-2 ATPase alters nucleosome remodelling properties in a mutation-specific manner. Nat Commun.

[65] Tong JK, Hassig CA, Schnitzler GR, Kingston RE, Schreiber SL (1998). Chromatin deacetylation by an ATP-dependent nucleosome remodelling complex. Nature.

[66] Xue Y, Wong J, Moreno GT, Young MK, Cote J, Wang W (1998). NURD, a novel complex with both ATP-dependent chromatin-remodeling and histone deacetylase activities. Mol Cell.

[67] Basta J, Rauchman M (2015). The nucleosome remodeling and deacetylase complex in development and disease. Transl Res.

[68] Ha M, Kraushaar DC, Zhao K (2014). Genome-wide analysis of H3.3 dissociation reveals high nucleosome turnover at distal regulatory regions of embryonic stem cells. Epigenetics Chromatin.

[69] Marques JG, Gryder BE, Pavlovic B, Chung Y, Ngo QA, Frommelt F, et al. NuRD subunit CHD4 regulates super-enhancer accessibility in rhabdomyosarcoma and represents a general tumor dependency. Elife. 2020;9. https://pubmed.ncbi.nlm.nih.gov/32744500/. 10.7554/eLife.54993 PMC7438112 32744500

[70] Jiao F, Li Z, He C, Xu W, Yang G, Liu T, Shen H, Cai J, Anastas JN, Mao Y (2020). RACK7 recognizes H3.3G34R mutation to suppress expression of MHC class II complex components and their delivery pathway in pediatric glioblastoma. Sci Adv.

[71] Gong F, Clouaire T, Aguirrebengoa M, Legube G, Miller KM (2017). Histone demethylase KDM5A regulates the ZMYND8-NuRD chromatin remodeler to promote DNA repair. J Cell Biol.

[72] Ghosh K, Tang M, Kumari N, Nandy A, Basu S, Mall DP, Rai K, Biswas D (2018). Positive regulation of transcription by human ZMYND8 through its association with P-TEFb complex. Cell Rep.

[73] Trizzino M, Barbieri E, Petracovici A, Wu S, Welsh SA, Owens TA, Licciulli S, Zhang R, Gardini A (2018). The tumor suppressor ARID1A controls global transcription via pausing of RNA polymerase II. Cell Rep.

[74] Bottardi S, Mavoungou L, Pak H, Daou S, Bourgoin V, Lakehal YA, Affar el B, Milot E (2014). The IKAROS interaction with a complex including chromatin remodeling and transcription elongation activities is required for hematopoiesis. PLoS Genet.

[75] Schwabish MA, Struhl K (2007). The Swi/Snf complex is important for histone eviction during transcriptional activation and RNA polymerase II elongation in vivo. Mol Cell Biol.

[76] Park JH, Park EJ, Lee HS, Kim SJ, Hur SK, Imbalzano AN, Kwon J (2006). Mammalian SWI/SNF complexes facilitate DNA double-strand break repair by promoting gamma-H2AX induction. EMBO J.

[77] Smeenk G, Wiegant WW, Vrolijk H, Solari AP, Pastink A, van Attikum H (2010). The NuRD chromatin-remodeling complex regulates signaling and repair of DNA damage. J Cell Biol.

[78] Whyte WA, Orlando DA, Hnisz D, Abraham BJ, Lin CY, Kagey MH, Rahl PB, Lee TI, Young RA (2013). Master transcription factors and mediator establish super-enhancers at key cell identity genes. Cell.

[79] Hazan I, Monin J, Bouwman BAM, Crosetto N, Aqeilan RI (2019). Activation of oncogenic super-enhancers is coupled with DNA repair by RAD51. Cell Rep.

[80] Martin M (2011). Cutadapt removes adapter sequences from high-throughput sequencing reads. EMBnet J.

[81] FastQC: a quality control tool for high throughput sequence data. http://www.bioinformatics.babraham.ac.uk/projects/fastqc.

[82] Ewels P, Magnusson M, Lundin S, Kaller M (2016). MultiQC: summarize analysis results for multiple tools and samples in a single report. Bioinformatics.

[83] Dobin A, Davis CA, Schlesinger F, Drenkow J, Zaleski C, Jha S, Batut P, Chaisson M, Gingeras TR (2013). STAR: ultrafast universal RNA-seq aligner. Bioinformatics.

[84] Love MI, Huber W, Anders S (2014). Moderated estimation of fold change and dispersion for RNA-seq data with DESeq2. Genome Biol.

[85] Love MI, Anders S, Kim V, Huber W (2015). RNA-Seq workflow: gene-level exploratory analysis and differential expression. F1000Res.

[86] Ignatiadis N, Klaus B, Zaugg JB, Huber W (2016). Data-driven hypothesis weighting increases detection power in genome-scale multiple testing. Nat Methods.

[87] Zhu A, Ibrahim JG, Love MI (2019). Heavy-tailed prior distributions for sequence count data: removing the noise and preserving large differences. Bioinformatics.

[88] Cornett EM, Dickson BM, Rothbart SB. Analysis of histone antibody specificity with peptide microarrays. J Vis Exp. 2017;126. https://pubmed.ncbi.nlm.nih.gov/28809825/. 10.3791/55912 PMC5613814 28809825

[89] Dickson BM, Cornett EM, Ramjan Z, Rothbart SB (2016). ArrayNinja: an open source platform for unified planning and analysis of microarray experiments. Methods Enzymol.

[90] Schneider VA, Graves-Lindsay T, Howe K, Bouk N, Chen HC, Kitts PA, Murphy TD, Pruitt KD, Thibaud-Nissen F, Albracht D (2017). Evaluation of GRCh38 and de novo haploid genome assemblies demonstrates the enduring quality of the reference assembly. Genome Res.

[91] Langmead B, Salzberg SL (2012). Fast gapped-read alignment with Bowtie 2. Nat Methods.

[92] Li H, Handsaker B, Wysoker A, Fennell T, Ruan J, Homer N, Marth G, Abecasis G, Durbin R, Genome Project Data Processing S (2009). The Sequence Alignment/Map format and SAMtools. Bioinformatics.

[93] Ou J, Liu H, Yu J, Kelliher MA, Castilla LH, Lawson ND, Zhu LJ (2018). ATACseqQC: a Bioconductor package for post-alignment quality assessment of ATAC-seq data. BMC Genomics.

[94] Daley T, Smith AD (2013). Predicting the molecular complexity of sequencing libraries. Nat Methods.

[95] Zhang Y, Liu T, Meyer CA, Eeckhoute J, Johnson DS, Bernstein BE, Nusbaum C, Myers RM, Brown M, Li W (2008). Model-based analysis of ChIP-Seq (MACS). Genome Biol.

[96] Amemiya HM, Kundaje A, Boyle AP (2019). The ENCODE Blacklist: identification of problematic regions of the genome. Sci Rep.

[97] Landt SG, Marinov GK, Kundaje A, Kheradpour P, Pauli F, Batzoglou S, Bernstein BE, Bickel P, Brown JB, Cayting P (2012). ChIP-seq guidelines and practices of the ENCODE and modENCODE consortia. Genome Res.

[98] Quinlan AR, Hall IM (2010). BEDTools: a flexible suite of utilities for comparing genomic features. Bioinformatics.

[99] Lun AT, Smyth GK (2016). csaw: a Bioconductor package for differential binding analysis of ChIP-seq data using sliding windows. Nucleic Acids Res.

[100] Robinson MD, McCarthy DJ, Smyth GK (2010). edgeR: a Bioconductor package for differential expression analysis of digital gene expression data. Bioinformatics.

[101] Ernst J, Kellis M (2017). Chromatin-state discovery and genome annotation with ChromHMM. Nat Protoc.

[102] Fiziev P, Akdemir KC, Miller JP, Keung EZ, Samant NS, Sharma S, Natale CA, Terranova CJ, Maitituoheti M, Amin SB (2017). Systematic epigenomic analysis reveals chromatin states associated with melanoma progression. Cell Rep.

[103] Gorkin DU, Barozzi I, Zhao Y, Zhang Y, Huang H, Lee AY, Li B, Chiou J, Wildberg A, Ding B (2020). An atlas of dynamic chromatin landscapes in mouse fetal development. Nature.

[104] Ritchie ME, Phipson B, Wu D, Hu Y, Law CW, Shi W, Smyth GK (2015). limma powers differential expression analyses for RNA-sequencing and microarray studies. Nucleic Acids Res.

[105] Davis S, Meltzer PS (2007). GEOquery: a bridge between the Gene Expression Omnibus (GEO) and BioConductor. Bioinformatics.

[106] Barrett T, Wilhite SE, Ledoux P, Evangelista C, Kim IF, Tomashevsky M, Marshall KA, Phillippy KH, Sherman PM, Holko M (2013). NCBI GEO: archive for functional genomics data sets--update. Nucleic Acids Res.

[107] Smedley D, Haider S, Ballester B, Holland R, London D, Thorisson G, Kasprzyk A (2009). BioMart--biological queries made easy. BMC Genomics.

[108] Heinz S, Benner C, Spann N, Bertolino E, Lin YC, Laslo P, Cheng JX, Murre C, Singh H, Glass CK (2010). Simple combinations of lineage-determining transcription factors prime cis-regulatory elements required for macrophage and B cell identities. Mol Cell.

[109] Lawrence M, Huber W, Pages H, Aboyoun P, Carlson M, Gentleman R, Morgan MT, Carey VJ (2013). Software for computing and annotating genomic ranges. PLoS Comput Biol.

[110] Robinson JT, Thorvaldsdottir H, Winckler W, Guttman M, Lander ES, Getz G, Mesirov JP (2011). Integrative genomics viewer. Nat Biotechnol.

[111] Gu Z, Eils R, Schlesner M (2016). Complex heatmaps reveal patterns and correlations in multidimensional genomic data. Bioinformatics.

[112] Wickham H (2016). ggplot2: Elegant Graphics for Data Analysis.

[113] R Core Team (2018). R: a language and environment for statistical computing.

[114] Reske JJ, Chandler RL. ARID1A-dependent maintenance of H3.3 is required for repressive CHD4-ZMYND8 chromatin interactions at super-enhancers. 2022, NCBI Gene Expression Omnibus, GSE190557. https://identifiers.org/geo:GSE190557. https://www.ncbi.nlm.nih.gov/geo/query/acc.cgi?acc=GSE190557. 10.1186/s12915-022-01407-y PMC9509632 36153585

[115] Wilson MR, Reske JJ, Chandler RL. ARID1A and PI3-Kinase pathway mutations in the endometrium drive epithelial transdifferentiation and collective invasion. 2019, NCBI Gene Expression Omnibus, GSE121198. https://identifiers.org/geo:GSE121198. https://www.ncbi.nlm.nih.gov/geo/query/acc.cgi?acc=GSE121198. 10.1038/s41467-019-11403-6 PMC6686004 31391455

[116] Wilson MR, Reske JJ, Chandler RL. ARID1A mutations promote P300-dependent endometrial invasion through super-enhancer hyperacetylation. 2020, NCBI Gene Expression Omnibus, GSE148474. https://identifiers.org/geo:GSE148474. https://www.ncbi.nlm.nih.gov/geo/query/acc.cgi?acc=GSE148474. 10.1016/j.celrep.2020.108366 PMC7682620 33176148

[117] Creighton C, Matzuk M, Hawkins S. Gene expression profiles of endometriosis. 2010, NCBI Gene Expression Omnibus, GSE23339. https://identifiers.org/geo:GSE23339. https://www.ncbi.nlm.nih.gov/geo/query/acc.cgi?acc=GSE23339.

