# Supplementary material for: ARID1A-dependent maintenance of H3.3 is required for repressive CHD4-ZMYND8 chromatin interactions at super-enhancers
Source: BMC Biol. 2022 Sep 25;20:209. doi: 10.1186/s12915-022-01407-y (PMC9509632; doi:10.1186/s12915-022-01407-y)
Supplement: Supplementary file 1 — Additional file 1: Supplementary Information. Figure S1. Supplemental ARID1A knockdown differential H3.3 data. Figure S2. H3.3 knockdown functional analysis. Figure S3. Effects of CHD4 loss on H3.3 chromatin and comparison to ARID1A. Figure S4. Additional chromatin features profiled in 12Z cells. Figure S5. Peptide specificity of anti-acetyl-H2A.Z (K4/K7). Figure S6. ChromHMM model optimization. Figure S7. Chromatin accessibility repressed by ARID1A is associated with H4 acetylation. Figure S8. Chromatin feature correlation across promoter-proximal super-enhancers. Figure S9. siCHD4/siZMYND8 functional analysis. Figure S10. H4K16ac enrichment at repressed mechanistic genes. Figure S11. Additive transcriptional repression by ARID1A and CHD4. Figure S12. Uncropped Western blots. [file 12915_2022_1407_MOESM1_ESM.pdf]

Additional file 1. Supplementary Information

Reske et al.

Figure S1

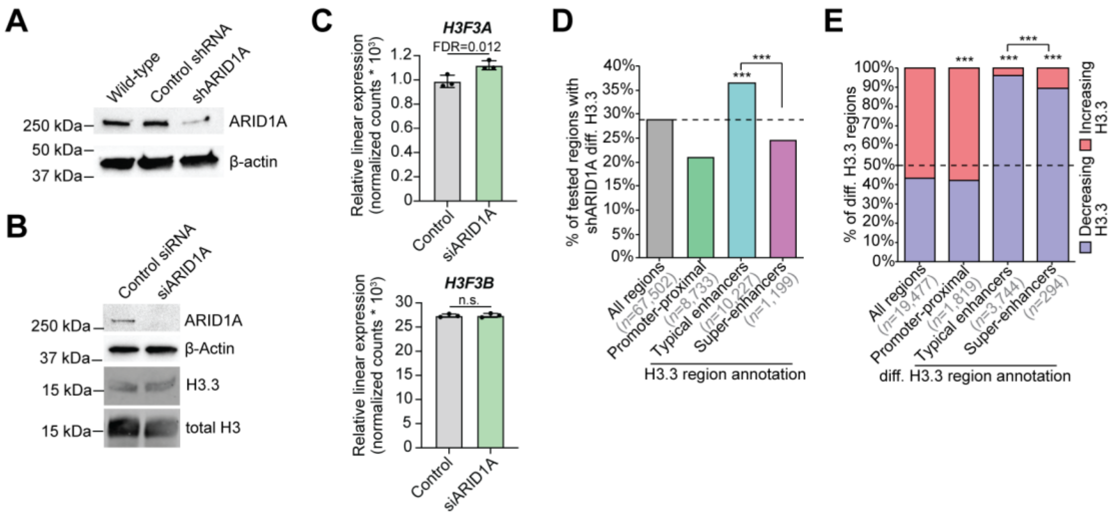

Fig. S1. Supplemental ARID1A knockdown differential H3.3 data. A, ARID1A immunoblot, compared to β-actin loading control, in 12Z cells treated with lentiviral shRNA particles to acutely knockdown ARID1A compared to non-targeting control shRNA. Wild-type condition shown as reference. **B**, Immunoblot for H3.3 and ARID1A, compared to total H3 and β-actin loading controls, respectively, in 12Z cells treated with siRNA to acutely knockdown ARID1A (siARID1A). H3.3 and total H3 were probed in the cellular histone fraction. **C**, RNA-seq linear gene expression data (normalized counts) for H3.3-encoding gene isoforms, *H3F3A* and *H3F3B*, following ARID1A knockdown by siRNA (siARID1A). **D**, Enrichment of regions displaying shARID1A significant differential H3.3 abundance among promoter-proximal regions, typical enhancers, and super-enhancers compared to all tested H3.3 regions. Gene promoter-proximal regions are defined as within 3 kb of a TSS. Enhancers are defined as ATAC+ H3K27ac peaks located >3 kb from a TSS. Super-enhancers were further distinguished from typical enhancers by *ROSE*. Statistics are hypergeometric enrichment and pairwise two-tailed Fisher's exact test. **E**, Distribution of significantly increasing vs. decreasing genomic H3.3 with ARID1A knockdown

among the classes described in **C**. Statistics are hypergeometric enrichment and pairwise two-tailed Fisher's exact test.

Figure S2

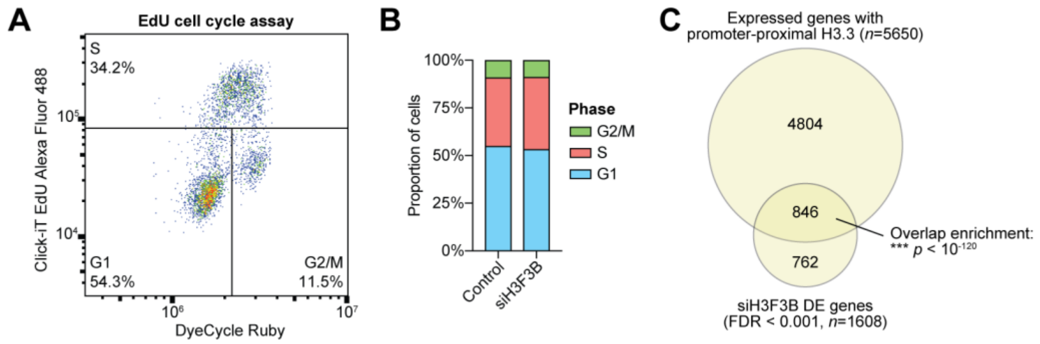

Fig. S2. H3.3 knockdown functional analysis. A, Representative cell cycle analysis by EdU labeling and DyeCycle ruby staining following 72 hours siRNA transfection. **B**, Quantification of cell cycle phases (G1, S, and G2/M) in control non-targeting siRNA treated and siH3F3B (H3.3 knockdown) treated 12Z cells ($n = 3$, pooled quantification). **C**, Euler diagram displaying overlap of genes with detected promoter-proximal H3.3 by ChIP-seq (*MACS2*, FDR < 0.05) and genes with altered expression following H3.3 knockdown by RNA-seq (*DESeq2*, FDR < 0.001). Statistic is hypergeometric enrichment.

Figure S3

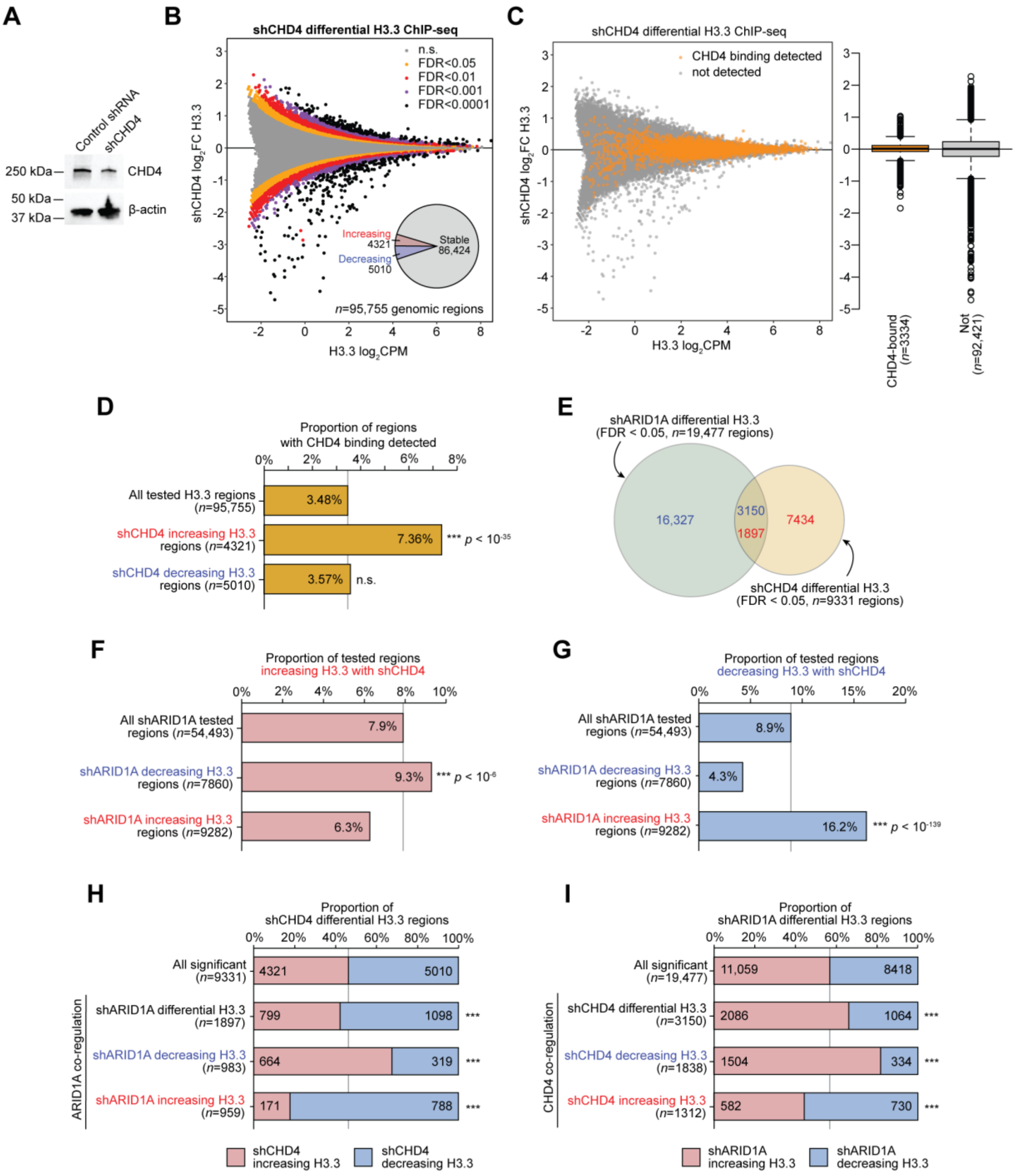

Fig. S3. Effects of CHD4 loss on H3.3 chromatin and comparison to ARID1A. A, CHD4 western blot, compared to β-actin loading control, in 12Z cells treated with lentiviral shRNA

particles to acutely knockdown CHD4. **B**, MA plot of differential H3.3 ChIP-seq ($n = 2$) in shCHD4 vs. control shRNA treated 12Z cells, across 95,755 tested genomic regions. Inset pie chart depicts distribution of significantly increasing and decreasing H3.3 regions (*csaw*/*edgeR* FDR < 0.05) compared to stable H3.3 (FDR > 0.05). Regions displaying FDR < 0.05 (*csaw*/*edgeR*) were interpreted as significant for downstream analyses. **C**, Segregation of shCHD4 differential H3.3 analysis by detected CHD4 binding in wild-type cells. Left, MA plot as in **B** but colored by CHD4 binding status. Right, boxplot quantifying the shCHD4 log_2FC H3.3 distribution among CHD4 bound vs. not bound tested regions. **D**, Enrichment for CHD4 binding detection among shCHD4 increasing ($n = 4321$) vs. decreasing ($n = 5010$) H3.3 regions compared to all tested regions. Statistic is hypergeometric enrichment. **E**, Overlap of regions displaying significant differential H3.3 abundance between shARID1A and shCHD4 conditions. **F**, Enrichment for shCHD4 increasing H3.3 among shARID1A differential H3.3 regions. Statistic is hypergeometric enrichment. **G**, Enrichment for shCHD4 decreasing H3.3 among shARID1A differential H3.3 regions. Statistic is hypergeometric enrichment. **H**, Distribution of significant shCHD4 increasing vs. decreasing regions functionally co-regulated by ARID1A (i.e. also differential with shARID1A). Statistic is hypergeometric enrichment. **I**, Distribution of significant shARID1A increasing vs. decreasing regions functionally co-regulated by CHD4 (i.e. also differential with shCHD4). Statistic is hypergeometric enrichment.

Figure S4

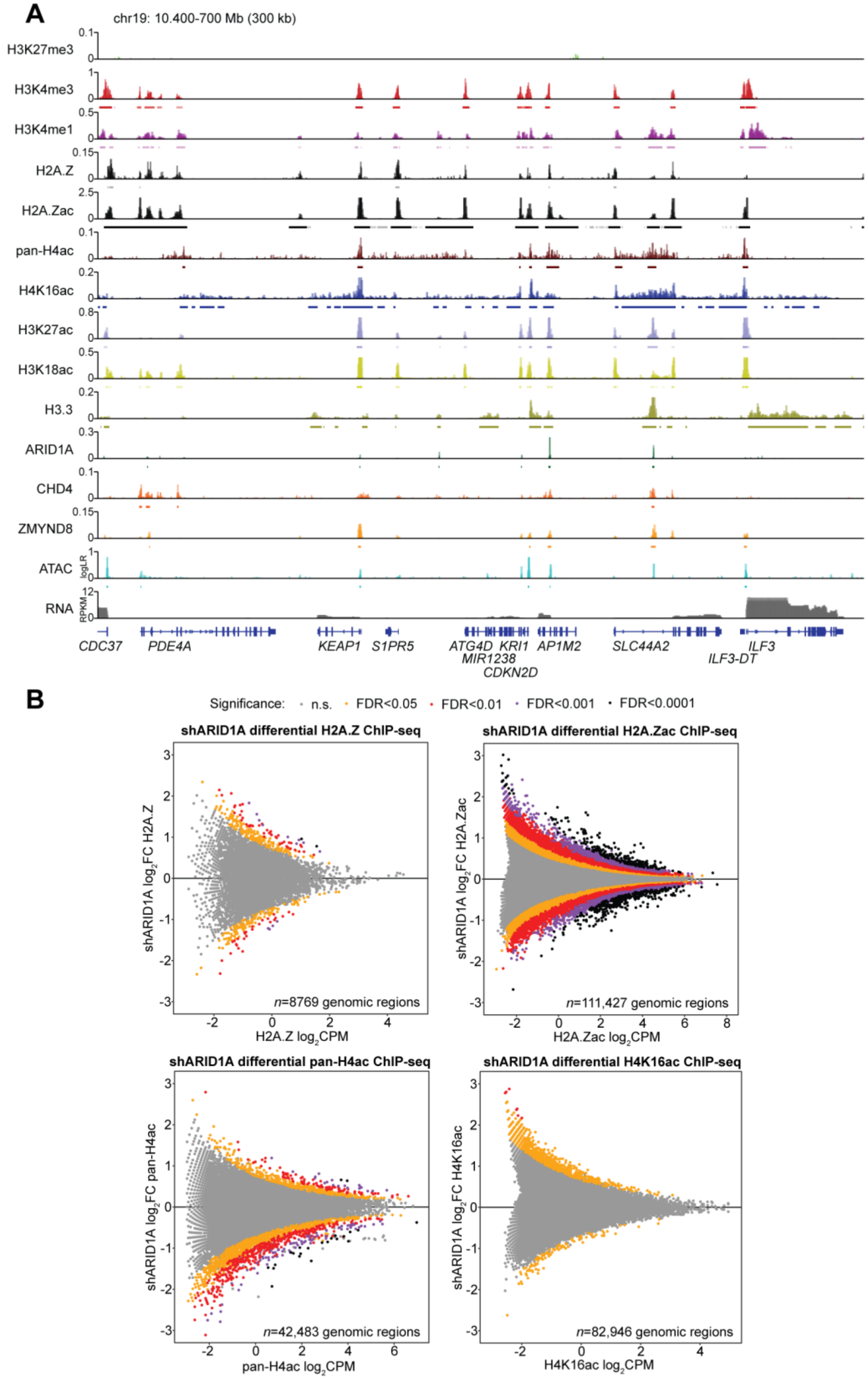
A
chr19: 10.400-700 Mb (300 kb)
H3K27me3
H3K4me3
H3K4me1
H2A.Z
H2A.Zac
pan-H4ac
H4K16ac
H3K27ac
H3K18ac
H3.3
ARID1A
CHD4
ZMYND8
ATAC
logLR
RNA
RPKM
CDC37
PDE4A
KEAP1
S1PR5
ATG4D
KRI1
MIR1238
CDKN2D
AP1M2
SLC44A2
ILF3
ILF3-DT
B
Significance: n.s. FDR<0.05 FDR<0.01 FDR<0.001 FDR<0.0001
shARID1A differential H2A.Z ChIP-seq
shARID1A log2FC H2A.Z
n=8769 genomic regions
H2A.Z log2CPM
shARID1A differential H2A.Zac ChIP-seq
shARID1A log2FC H2A.Zac
n=111,427 genomic regions
H2A.Zac log2CPM
shARID1A differential pan-H4ac ChIP-seq
shARID1A log2FC pan-H4ac
n=42,483 genomic regions
pan-H4ac log2CPM
shARID1A differential H4K16ac ChIP-seq
shARID1A log2FC H4K16ac
n=82,946 genomic regions
H4K16ac log2CPM

Fig. S4. Additional chromatin features profiled in 12Z cells. A, Example hg38 locus on chromosome 19 depicting all analyzed chromatin features in this study. *y*-axis is log-likelihood ratio (logLR) of assay signal (compared to input chromatin for ChIP-seq or background genome for ATAC-seq) or RPKM for total RNA. Small bars under tracks indicate significant peak detection by *MACS2* (FDR < 0.05). **B**, MA plots for new shARID1A vs. control differential ChIP-seq ($n = 2$) experiments: H2A.Z, H2A.Zac (K4/K7), pan-H4ac (K5/K8/K12/K16), and H4K16ac. Genomic regions are colored by significance (*csaw*/*edgeR*).

Figure S5

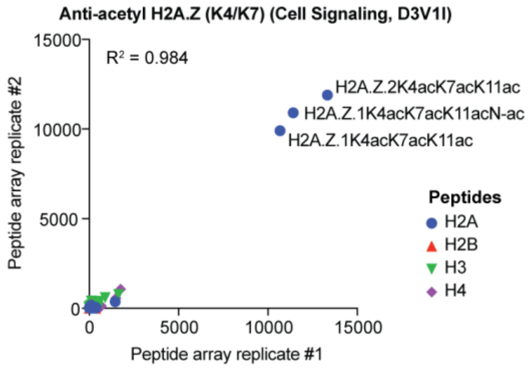

Fig. S5. Peptide specificity of anti-acetyl-H2A.Z (K4/K7). Hybridization results for two independent histone peptide microarray replicates probed with anti-acetyl-H2A.Z (H2A.Zac) (Cell Signaling, D3V1I), demonstrating clear specificity for acetylated H2A.Z peptides. The three dominantly detected H2A.Zac peptides are labeled.

Figure S6

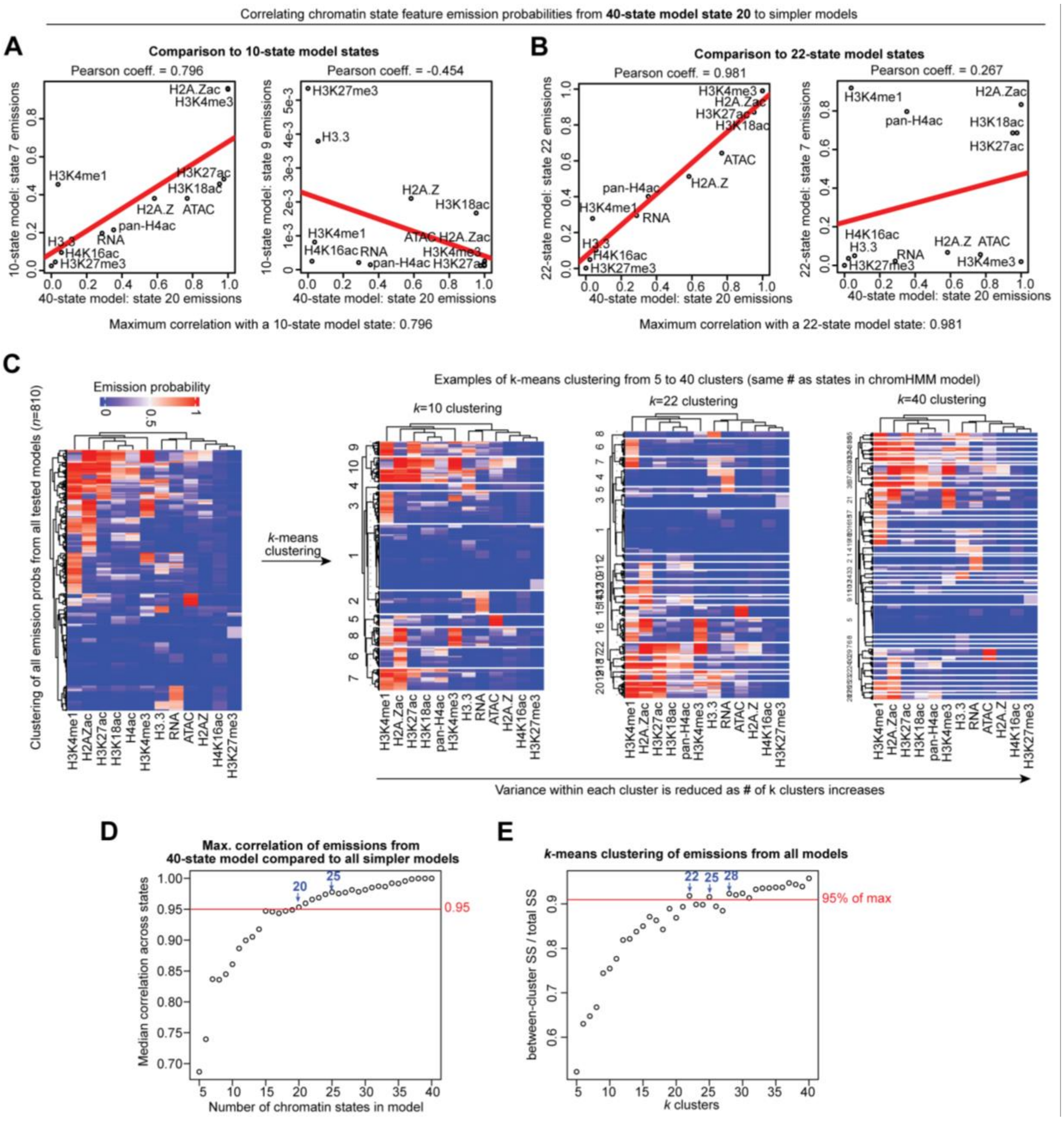

Fig. S6. ChromHMM model optimization. Examples and results of quantitative strategies used to select optimal ChromHMM model as devised by Gorkin et al. See Methods for further details and procedural description. **A**-**B**, Correlating chromatin state feature emission probabilities from the most complex model (40-state) to all simpler models. **A**, Comparison to 10-state model states.

The maximum Pearson correlation coefficient of 40-state model state 20 with a 10-state model state is 0.796. **B**, Comparison to 22-state model states. The maximum Pearson correlation coefficient of 40-state model state 20 with a 22-state model state is 0.981. **C**, Clustered heatmaps of all emission probabilities from all tested models (left, $n = 810$ total modeled chromatin states), followed by examples of k-means clustering (right), k-means clustering was performed from $k = 5$ to $k = 40$ clusters (the same number of states in the tested chromatin state models) for measurements of goodness of fit by sum-of-squares (between-cluster vs. total). Variance within each cluster is reduced as the number of k clusters increases. **D**, First strategy model selection plot (as in **A**-**B**) based on the maximum correlation of emissions from the most complex model (40-state) compared to all simpler models. The x-axis is the simpler model used for comparison, and the y-axis is the median of maximum correlation coefficients across all 40-state model states compared to states within the simpler model. 95% threshold is represented by the red line. **E**, Second strategy model selection plot (as in **C**) based on the goodness of fit for all simpler models relative to the most complex model (40-state). The y-axis is the amount of variance explained by clustering, i.e. the ratio of between-cluster sum-of-squares to total sum-of-squares, relative to k = 40 clusters (the number of chromatin states in the most complex model). 95% threshold is represented by the red line.

Figure S7

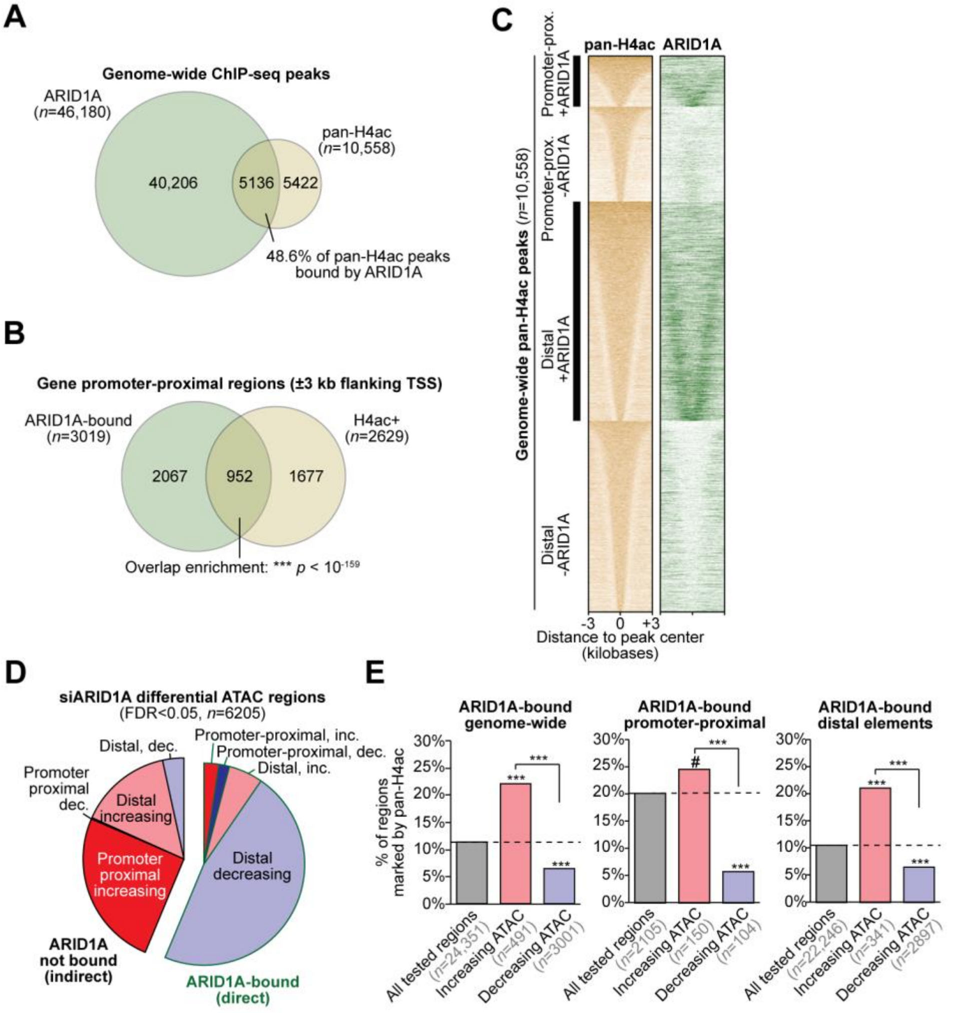

Fig. S7. Chromatin accessibility repressed by ARID1A is associated with H4 acetylation. A, Euler diagram displaying overlapping genome-wide ChIP-seq peaks (called by *MACS2*) for pan-H4ac compared to ARID1A. 48.6% of pan-H4ac peaks are co-marked by ARID1A binding. **B**, Euler diagram displaying overlapping gene promoters marked by pan-H4ac (H4ac+) and ARID1A binding. Statistic is hypergeometric enrichment test. **C**, Heatmap displaying ChIP-seq signal across 10,558 genome-wide pan-H4ac ChIP-seq peaks. Signal is quantified as ChIP – Input. Peaks are ranked by overall pan-H4ac signal and stratified by ARID1A binding and promoter (<3 kb from a TSS) vs. distal (>3 kb from a TSS). **D**, Annotation and directional breakdown of 6205

significant (FDR < 0.05) differentially accessible genomic regions, measured by ATAC-seq, following ARID1A depletion via siRNA (siARID1A). Regions are further segregated based on ARID1A binding status. **E**, Association of pan-H4ac with siARID1A differential ATAC regions directly bound by ARID1A and separated by direction of accessibility change genome-wide (left), at promoters (center), and at distal elements (right). Statistic is hypergeometric enrichment test and two-tailed Fisher's exact test. # $p < 0.10$, *** $p < 0.001$.

Figure S8

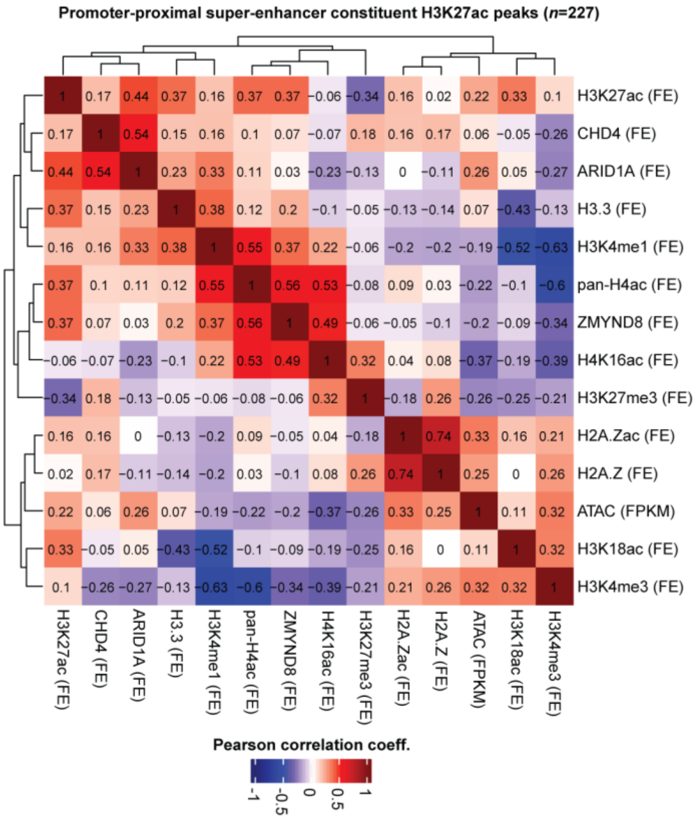

Fig. S8. Chromatin feature correlation across promoter-proximal super-enhancers. Clustered heatmap of Pearson correlation coefficients for all measured chromatin features (aside from total RNA) across 227 promoter-proximal super-enhancer constituent H3K27ac peaks. These active super-enhancer-like regions (distinguished by *ROSE*) overlap the 3 kb promoter region flanking a gene TSS. ChIP-seq assays are quantified as ChIP/input chromatin fold-enrichment (FE), and ATAC-seq is quantified as FPKM.

Figure S9

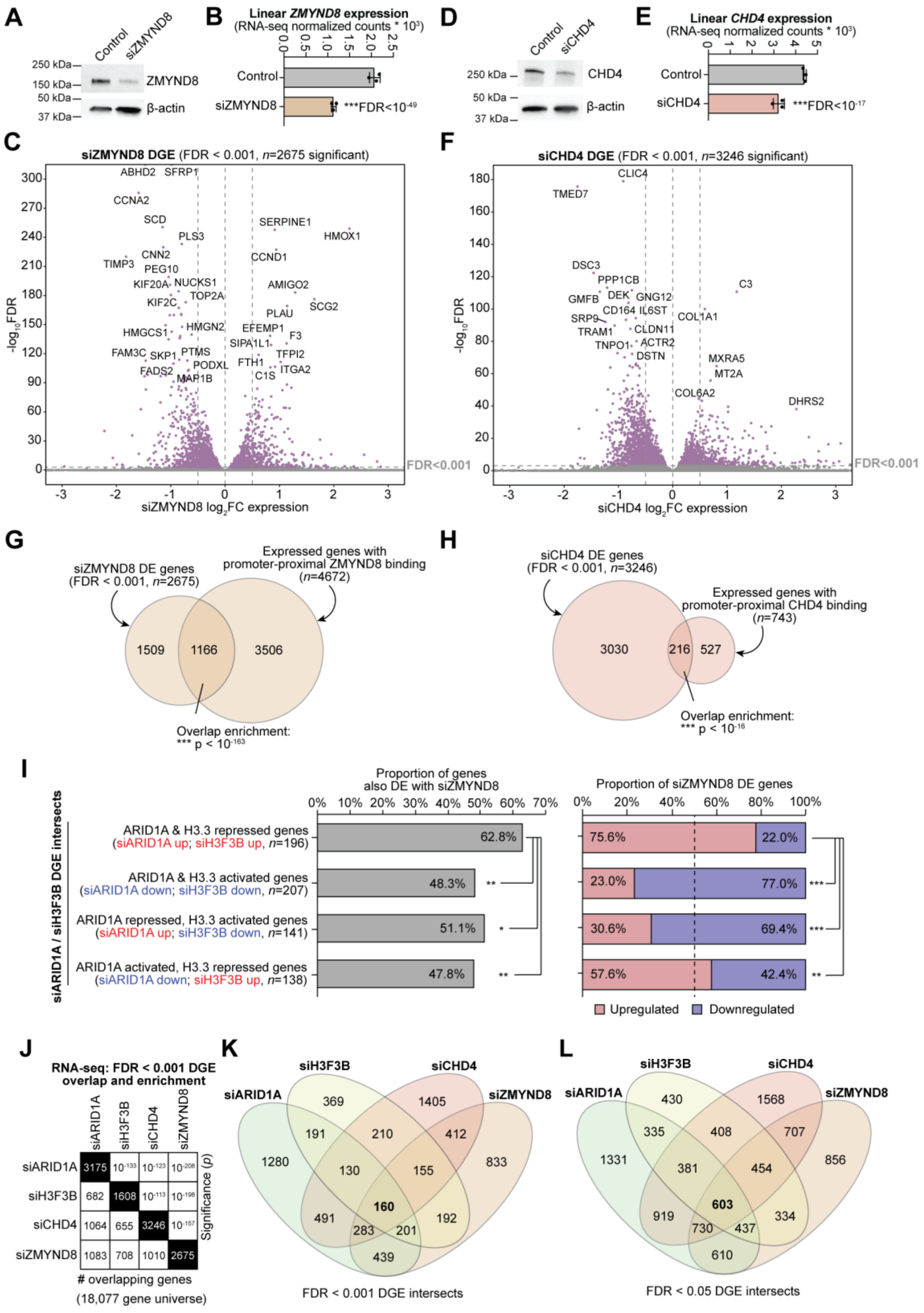

A
Control
siZMYND8
ZMYND8
β-actin
B
Linear ZMYND8 expression
(RNA-seq normalized counts * 10^3)
Control
siZMYND8
***FDR<10^-49
D
Control
siCHD4
CHD4
β-actin
E
Linear CHD4 expression
(RNA-seq normalized counts * 10^3)
Control
siCHD4
***FDR<10^-17
C
siZMYND8 DGE (FDR < 0.001, n=2675 significant)
-log10FDR
siZMYND8 log2FC expression
FDR<0.001
F
siCHD4 DGE (FDR < 0.001, n=3246 significant)
-log10FDR
siCHD4 log2FC expression
FDR<0.001
G
siZMYND8 DE genes (FDR < 0.001, n=2675)
Expressed genes with promoter-proximal ZMYND8 binding (n=4672)
1509
1166
3506
Overlap enrichment: *** p < 10^-163
H
siCHD4 DE genes (FDR < 0.001, n=3246)
Expressed genes with promoter-proximal CHD4 binding (n=743)
3030
216
527
Overlap enrichment: *** p < 10^-16
I
siARID1A / siH3F3B DGE intersects
Proportion of genes also DE with siZMYND8
Proportion of siZMYND8 DE genes
ARID1A & H3.3 repressed genes (siARID1A up; siH3F3B up, n=196)
62.8%
75.6%
22.0%
ARID1A & H3.3 activated genes (siARID1A down; siH3F3B down, n=207)
48.3%
23.0%
77.0%
ARID1A repressed, H3.3 activated genes (siARID1A up; siH3F3B down, n=141)
51.1%
30.6%
69.4%
ARID1A activated, H3.3 repressed genes (siARID1A down; siH3F3B up, n=138)
47.8%
57.6%
42.4%
Upregulated
Downregulated
J
RNA-seq: FDR < 0.001 DGE overlap and enrichment
siARID1A
siH3F3B
siCHD4
siZMYND8
Significance (p)
overlapping genes
(18,077 gene universe)
K
siARID1A
siH3F3B
siCHD4
siZMYND8
FDR < 0.001 DGE intersects
L
siARID1A
siH3F3B
siCHD4
siZMYND8
FDR < 0.05 DGE intersects

Fig. S9. siCHD4/siZMYND8 functional analysis. A, Immunoblot for (top) ZMYND8 compared to (bottom) β-actin loading control in 12Z cells treated with non-targeting control siRNA or siZMYND8 (ZMYND8 knockdown). **B**, RNA-seq expression of ZMYND8 in control and siZMYND8 cells. Statistic is FDR-adjusted *DESeq2* Wald test. **C**, Volcano plot for RNA-seq differential gene expression between siZMYND8 and control cells (n = 3). FDR < 0.001 was used as a significance threshold. Top significant ZMYND8-dependent genes are labeled. **D**-**F**, CHD4 siRNA knockdown (siCHD4) framework and RNA-seq analysis (n = 3) as in **A**-**C**. **G**, Euler diagram displaying overlap between siZMYND8 differential gene expression (DGE) and ZMYND8 promoter-bound genes. Statistic is hypergeometric enrichment test. **H**, Euler diagram displaying overlap between siCHD4 DGE and CHD4 promoter-bound genes. Statistic is hypergeometric enrichment test. **I**, Left, association of siZMYND8 DGE among ARID1A-H3.3 co-regulated gene classes described in Fig. 3F-H. Right, distribution of significantly upregulated (ZMYND8 repressed) vs. downregulated (ZMYND8 activated) siZMYND8 DE genes among ARID1A-H3.3 co-regulated gene classes. Statistic is two-tailed Fisher's exact test. **J**, RNA-seq DGE (FDR < 0.001) overlap and enrichment across the four analyzed knockdown conditions: siARID1A, siH3F3B, siCHD4, and siZMYND8. The black cell diagonal represents the number of total significant DE genes in that condition. The bottom-left triangle displays the number of overlapping DE genes between pairwise knockdowns. The upper-right triangle displays the overlap enrichment significance by hypergeometric enrichment test. A reduced 18,077 gene set universe was used that contains genes with detected expression in all analyzed conditions. **K**, Overlap of FDR < 0.001 DGE sets across the four analyzed knockdown conditions. **L**, Overlap of FDR < 0.05 DGE sets across the four analyzed knockdown conditions, corresponding with Fig. 7F.

Figure S10

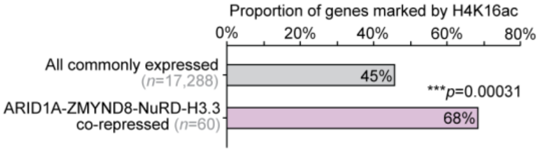

Fig. S10. H4K16ac enrichment at repressed mechanistic genes. Enrichment of H4K16ac overlapping gene promoters or gene bodies of ARID1A-H3.3-ZMYND8-CHD4 co-repressed genes—i.e. genes that are upregulated (FDR < 0.05) following treatment with siARID1A, siH3F3B, siZMYND8, and siCHD4—compared to all expressed genes with chromatin data. Statistic is hypergeometric enrichment test.

Figure S11

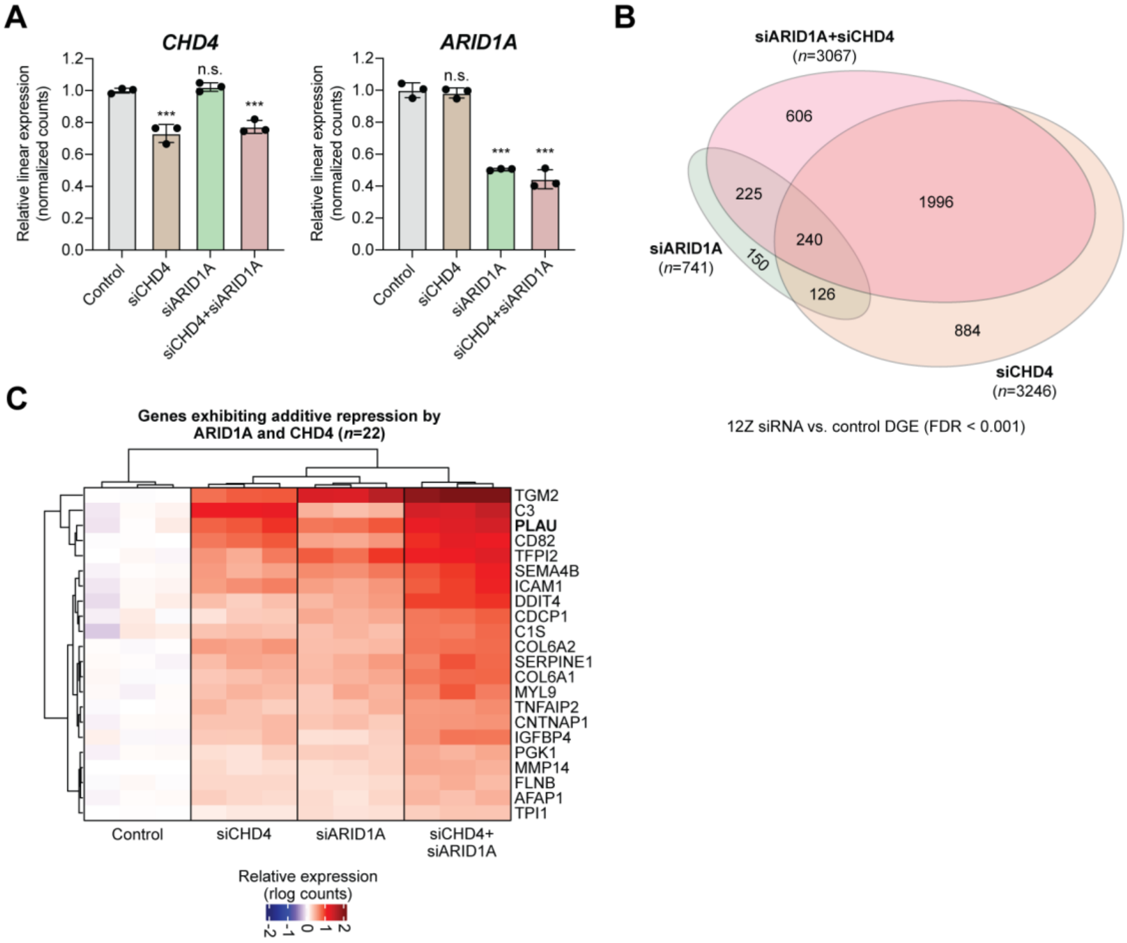

Fig. S11. Additive transcriptional repression by ARID1A and CHD4. RNA-seq analysis of an ARID1A ± CHD4 co-knockdown experiment (n = 3 per condition). **A**, Relative linear RNA-seq expression for (left) CHD4 and (right) ARID1A among siRNA treated conditions. Statistic is *DESeq2* FDR-adjusted Wald test. **B**, Euler diagram displaying DGE (FDR < 0.001) overlap of siCHD4, siARID1A, and siARID1A+siCHD4 conditions compared to control cells. **C**, Clustered heatmap of expression alterations (rlog) at 22 genes displaying additive repression by ARID1A and CHD4. These genes are significantly upregulated in siCHD4, siARID1A, and siARID1A+siCHD4 conditions compared to control cells and siARID1A+siCHD4 vs. single-knockdown conditions. *** FDR < 0.001

Figure S12

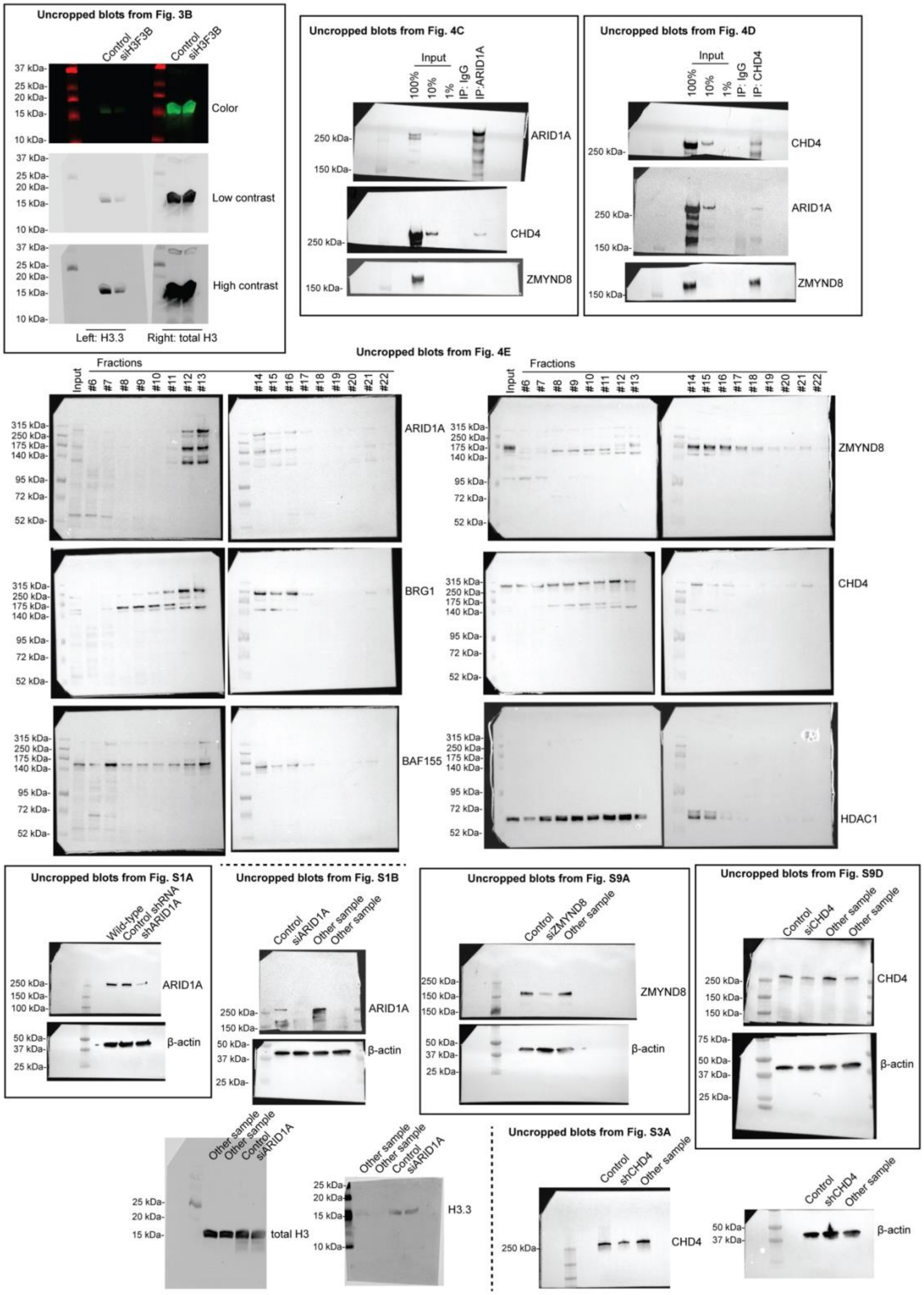

Fig. S12. Uncropped Western blots.
